# Identification and Characterization of Alcohol-related Hepatocellular Carcinoma Prognostic Subtypes based on an Integrative N6-methyladenosine methylation Model

**DOI:** 10.7150/ijbs.62168

**Published:** 2021-08-14

**Authors:** Yue Zhang, Fanhong Zeng, Min Zeng, Xu Han, Lei Cai, Jiajun Zhang, Jun Weng, Yi Gao

**Affiliations:** 1 Department of Hepatobiliary Surgery II, Guangdong Provincial Research Center for Artificial Organ and Tissue Engineering, Guangzhou Clinical Research and Transformation Center for Artificial Liver, Institute of Regenerative Medicine, ZhuJiang Hospital, Southern Medical University, Guangzhou, Guangdong Province, China.; 2 State Key Laboratory of Organ Failure Research, Southern Medical University, Guangzhou, China.

**Keywords:** Hepatocellular carcinoma, N6-methyladenosine, tumour immune microenvironment, treatment sensitivity, teniposide

## Abstract

**Background:** Alcohol consumption increases the risk of hepatocellular carcinoma (HCC), and associated with a high mortality rate and poor prognosis. N6-methyladenosine (m6A) methylations play key roles in tumorigenesis and progression. However, our current knowledge about m6A in alcohol-related HCC (A-HCC) remains elucidated. Herein, the authors construct an integrative m6A model based on A-HCC subtyping and mechanism exploration workflow.

**Methods:** Based on the m6A expressions of A-HCC and *in vivo* experiment, different prognosis risk A-HCC subtypes are identified. Meanwhile, multiple interdependent indicators of prognosis including patient survival rate, clinical pathological prognosis and immunotherapy sensitivity.

**Results:** The m6A model includes LRPPRC, YTHDF2, KIAA14219, and RBM15B, classified A-HCC patients into high/low-risk subtypes. The high-risk subtype compared to the low-risk subtype showed phenotypic malignancy, poor prognosis, immunosuppression, and activation of tumorigenesis and proliferation-related pathways, including the E2F target, DNA repair, and mTORC1 signalling pathways. The expression of Immunosuppressive cytokines DNMT1/EZH2 was up-regulated in A-HCC patients, and teniposide may be a potential therapeutic drug for A-HCC.

**Conclusion:** Our model redefined A-HCC prognosis risk, identified potential m6As linking tumour progress and immune regulations and selected possible therapy target, thus promoting understanding and clinical applications about A-HCC.

## Introduction

N6-Methyladenosine (m6A) methylation is the most common post-transcriptional modification, regulating RNA splicing, translocation, stability, and translation into proteins 1, 2. Enzymes involved in m6A methylation include demethylases (commonly referred as 'erasers'), methylases ('writers'), and methylation recognition enzymes ('readers') 3. Abnormal activity of m6A-related enzymes can induce numerous pathological responses, including tumour development, immune dysregulation, and embryonic developmental delay 4-6. Therefore, understanding the m6A alterations can provide meaningful insights into the tumorigenesis, progression and potential therapeutic targets.

Liver cancer is one of the most common cancers and the fourth leading cause of cancer-related deaths, with more than 850,000 annually reported cases worldwide 7-10. The most common risk factors for HCC are viral infection, alcoholism, and metabolic syndrome 11-13. Alcohol can significantly increase the release of cytoplasmic mtDNA, thereby activating the cGAS-IRF3 signal, causing liver cell apoptosis and inflammation 14, 15. Excessive alcohol intake induces a metabolism shift from oxidative metabolism to reductive reaction, which favour the synthesis of fatty acids and promote fatty liver progress, leading to liver cirrhosis and cancer 16, 17. In addition, chronic alcohol intake can cause reactive oxygen species and DNA damage, and further promote the activation of cancer stem cell-related gene mutations, leading to a poor prognosis for A-HCC 18, which has a mortality rate that is 4 times that of the general population 19.

The specific molecular mechanisms underlying A-HCC remain to be elucidated. The two most recognised major drivers are cytochrome P450 2E1 (*CYP2E1*) and intestinal lipopolysaccharide (LPS) imbalance 20, 21. Alcohol could induce liver inflammation and oxidative stress cause DNA damage in hepatocytes; ultimately promote tumour initiation and progression 22. Previously, m6A methylation was reported to play a promoting role in the occurrence and development of HCC, regulating cell proliferation, cell invasion and epithelial to mesenchymal transformation 23. The levels and activities of m6A regulatory genes YTHDF2, ALKBH5 and FTO can inhibit the HCC malignancy 24-26. For example, FTO can control liver energy homeostasis and metabolism, and it plays an anticancer role in the HCC development 27.

Here, to further explore the correlation between the level of m6A methylations and the occurrence and prognosis of A-HCC. We propose an integrative m6A model based on A-HCC subtyping and mechanism exploration workflow. Then, based on the m6A regulatory factors and multi-omics data from the cancer genome atlas (TCGA) two A-HCC subtypes and their corresponding biological and clinical characters were identified. We observed high-risk A-HCC subtypes are related to immunosuppression and some key Immunosuppressive cytokines (EZH2 and DNMT1) promote the poor prognosis of A-HCC patients. In addition, we selected possible therapy target, thereby promoting a comprehensive understanding of A-HCC and providing guidelines for its treatment.

## Materials and Methods

### Patients and specimens

For this study, we collected samples from 108 patients who underwent a liver biopsy at Zhujiang Hospital (Southern Medical University, Guangzhou, China) between 2018 and February 2021. After formalin fixation for 24 h, the samples were dehydrated, embedded in paraffin, and stored at 4 °C. The samples were divided into three groups: normal (no HCC history, n = 31), N-A-HCC (no history of alcohol consumption, n = 56), and A-HCC (history of alcohol consumption for more than 20 years, n = 21) (Supplementary Table 1). The data and tissue samples used in this study met the medical ethical requirements of the Southern Medical University.

### Mice, diets, and experimental design

C57BL/6 mice were obtained from the Guangdong Animal Experiment Center, China and they were kept in a specific pathogen-free environment at a constant temperature and light-dark cycle of 12 h. All animal handling procedures were approved by the Southern Medical University Animal Care and Use Committee.

To establish a tumour model, C57BL/6 mice were intraperitoneal injected with 25 mg/kg diethylnitrosamine (DEN; Sigma, USA) at 2 weeks of age. At 6 months, mice injected with DEN were given a non-alcoholic liquid diet for diet adaption. One week later, the experimental group was switched to an alcoholic liquid diet (the alcohol concentration was gradually increased to 4.8%), while the control group continued to receive a non-alcoholic liquid diet (maltose instead of alcohol with the same caloric content). Mice in the experimental treatment group were given teniposide (0.4 mg/d per kg bodyweight; Topscience, China) via gavage for 8 weeks at 6 months of age.

### Cell culture and reagents

The human HCC-derived cell lines Huh7 and HepG2 were obtained from the American Type Culture Collection (ATCC, Manassas, VA, USA). Cells were cultured in Dulbecco's modified Eagle's medium (DMEM; Gibco, USA) supplemented with 10% foetal bovine serum (FBS; Gibco) and 1% penicillin/streptomycin (Gibco) at 37 °C in a 5% CO_2_ atmosphere. Mid-log phase cells were used in all experiments.

When the cells achieved the desired confluency, the cells were starved by culturing in medium without FBS for 24 h. Subsequently, the cells were incubated with medium containing 100 mM ethanol for 48 h at 37 °C in a 5% CO_2_ humidified environment. These cells were used as *in vitro* model of A-HCC. Then, the cells were incubated for 12 h with 0.5 μM teniposide (Alexis Biochemicals, San Diego, CA, USA).

### Quantitative reverse transcription PCR (qRT-PCR)

Total RNA was extracted from clinical patient samples and cells using TRIzol reagent (Life Technologies, USA). RNA concentration and quality were measured using a spectrophotometer (Nanodrop One, Thermo Fisher Scientific, Waltham, MA, USA). Samples with a 260/280 absorbance ratio > 2 ± 0.1 were considered contaminated with protein and discarded. RNA samples were then reverse transcribed to cDNA using a reverse transcription kit (#RR037A; Takara Bio, Shiga, Japan), after which qPCR was performed using SYBR Premix Ex Taq (DRR041A; Takara Bio). Specific primers as shown in Supplementary Table 2 were used to detect the expression levels of relevant genes.

### Immunohistochemistry and immunofluorescence

To perform immunohistochemistry (IHC) on patient liver samples, the samples were processed into 4 μm-thick paraffin sections, deparaffinized, and hydrated, followed by microwave treatment (10 mM citrate buffer) for antigen retrieval. The tissue sections were treated with 3% H2O2 for 15 min to block endogenous peroxidase and with goat serum to prevent nonspecific antibody binding. Thereafter, they were incubated overnight at 4 °C with the primary antibodies against DNMT1 (ab188453; Abcam, Cambridge, England), EZH2 (ab191080; Abcam), KIAA1429 (PA5-95717, Thermo Fisher Scientific), LRPPRC (sc-166178,Santa Cruz Biotechnology, Dallas, TX, USA), RBM15B (PA5-110279, Thermo Fisher Scientific, USA) and YTHDF2 (PA5-100053, Thermo Fisher Scientific), followed by incubation with the secondary antibody at room temperature for 1h. For IHC staining, 3,3-diaminobenzidine (DAB; DA1010; Solarbio, China) was used and cell nuclei were counterstained with haematoxylin. Tissue sections were observed using brightfield microscopy.

For immunofluorescence, the cells were fixed with 4% paraformaldehyde, incubated with Triton, blocked with goat serum, and incubated with primary antibodies against DNMT1 and EZH2 at 4 °C overnight and with secondary antibodies (ab150077; Abcam) at room temperature for 1 h. The nuclei were counterstained with DAPI, after which the samples were imaged using a fluorescence microscope.

### Western blotting

Cultured cells were dissolved in RIPA buffer containing protease and phosphatase inhibitors. Proteins were collected by centrifugation (10,000 rpm for 10 min) and their concentrations determined using the bicinchoninic acid assay (BCA; Thermo Fisher Scientific). Proteins were subjected to NuPAGE Bis-Tris Gel Electrophoresis (#NP0321; Invitrogen, USA), transferred to nitrocellulose membranes, and incubated with the corresponding antibodies against DNMT1 (1:1,000), EZH2 (1:500), and β-actin (1:1,000; ab8226; Abcam).

### Data acquisition

Tumour RNA-seq data and clinical information were obtained from two separate series of patients. The first series obtained from The Cancer Genome Atlas (TCGA, https://portal.gdc.cancer.gov/), included 167 samples (117 A-HCC samples and 50 normal liver samples), and was used as the training set. The second series consisted of 316 samples (114 A-HCC samples and 202 normal liver samples) from the International Cancer Genome Consortium (ICGC, https://icgc.org/) and was used as the validation set. The relationship between m6A-regulators was determined used the Search Tool for Retrieval of Interacting Genes/Proteins (STRING, https://string-db.org/). The clinical characteristics of each patient series are shown in Supplementary Tables 3 and 4. Data obtained from TCGA and ICGC databases are freely available to the public, and this research strictly followed access policies and publication guidelines. Therefore, this study did not require ethical review or approval from an Ethics Committee.

Genes related to KIAA1429, LRPPRC, RBM15B, and YTHDF2, as well as mutation data, were obtained from Cbioportal (http://www.cbioportal.org/). Co-expressed genes were considered those with a Spearman's coefficient greater than 0.3. For gene selection, the threshold parameters were R ≥ 0.3, and *p* < 0.05. Drug sensitivity data were obtained from the Cancer Therapeutics Response Portal (CTRP) database of GSCALite (http://bioinfo.life.hust.edu.cn/web/GSCALite/) 28. The Immune Cell Abundance Identifier (ImmunoCellAI, http://bioinfo.life.hust.edu.cn/ImmuCellAI#!/) tool was used to predict immunotherapy response 29. The relationship of 21 m6A regulators was downloaded from GeneMANIA (http://genemania.org/).

### Risk model constitution

The 21 m6A-regulators, comprising eight writers (METTL3, METTL14, RBM15, RBM15B, WTAP, KIAA1429, CBLL1, ZC3H13), two erasers (ALKBH5 and FTO), and 11 readers (YTHDC1, YTHDC2, YTHDF1, YTHDF2, YTHDF3, IGF2BP1, HNRNPA2B1, HNRNPC, FMR1, LRPPRC, ELAVL1), were selected based on a previous report 30. To quantify the effects of m6A-regulators, statistically significant m6A-regulators selected from univariable Cox regression were analysed using least absolute shrinkage and selection operator (LASSO) regression. Statistical significance was set at *p* < 0.05. The hazard ratios and 95% confidence intervals were calculated. A total of 11 m6A-regulators were selected for further analysis. The m6A-risk model was developed using the LASSO Cox regression algorithm. The applied formula was as follows:

Risk score =
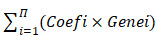


### Gene set enrichment analysis (GSEA)

GSEA analysis was performed using GSEA software (version 4.0.3) to detect the difference in enriched pathways between the high-risk and low-risk subtypes by the Molecular Signatures Database (MSigDB, h.all.v7.2.symbols.gmt). For each analysis, gene set permutations were performed 1,000 times.

### Estimation of immune cell type

We used the single-sample GSEA (ssGSEA) algorithm to quantify the relative abundance of infiltrated immune cells. The gene set stores a variety of human immune cell subtypes, including T cells, dendritic cells, macrophages, and B cells 31, 32. The enrichment score calculated using ssGSEA analysis was used to assess infiltrated immune cells in each sample.

### Statistical analysis

Relationships among the m6A regulators were calculated using Pearson's correlation based on gene expression. Continuous variables are summarised as mean ± standard deviation (SD). Differences between groups were compared using the Wilcoxon test, using the R software. Different m6A-risk subtypes were compared using the Kruskal-Wallis test. The 'ConsensusClusterPlus' package in R was used for consistent clustering to determine the subgroup of A-HCC samples from TCGA. The Euclidean squared distance metric and K-means clustering algorithm were used to divide the sample from k = 2 to k = 9. Approximately 80% of the samples were selected in each iteration, and the results were obtained after 100 iterations 33. The optimal number of clusters was determined using a consistent cumulative distribution function graph. Thereafter, the results were depicted as heatmaps of the consistency matrix generated by the 'heatmap' R package.

We then used Kaplan-Meier analysis to compare the disease-specific survival (DSS), disease-free interval (DFI), progression-free interval (PFI) or overall survival (OS) between different subtypes using the 'survival' and 'survminer' packages in R software. The significance of differences in survival time was calculated using the log-rank test with a threshold of *p* < 0.05. Univariate and multivariate analyses were performed using Cox regression, followed by identification of independent risk factors for DSS, DFI, PFI, and OS in A-HCC. To evaluate the accuracy and sensibility of the model, we constructed the receiver operating characteristic (ROC) curve and calculated the area under the curve (AUC) using the 'survivalROC' package in R software.

## Results

### Regulatory pattern of m6A-related genes in A-HCC

**Figure 1:**
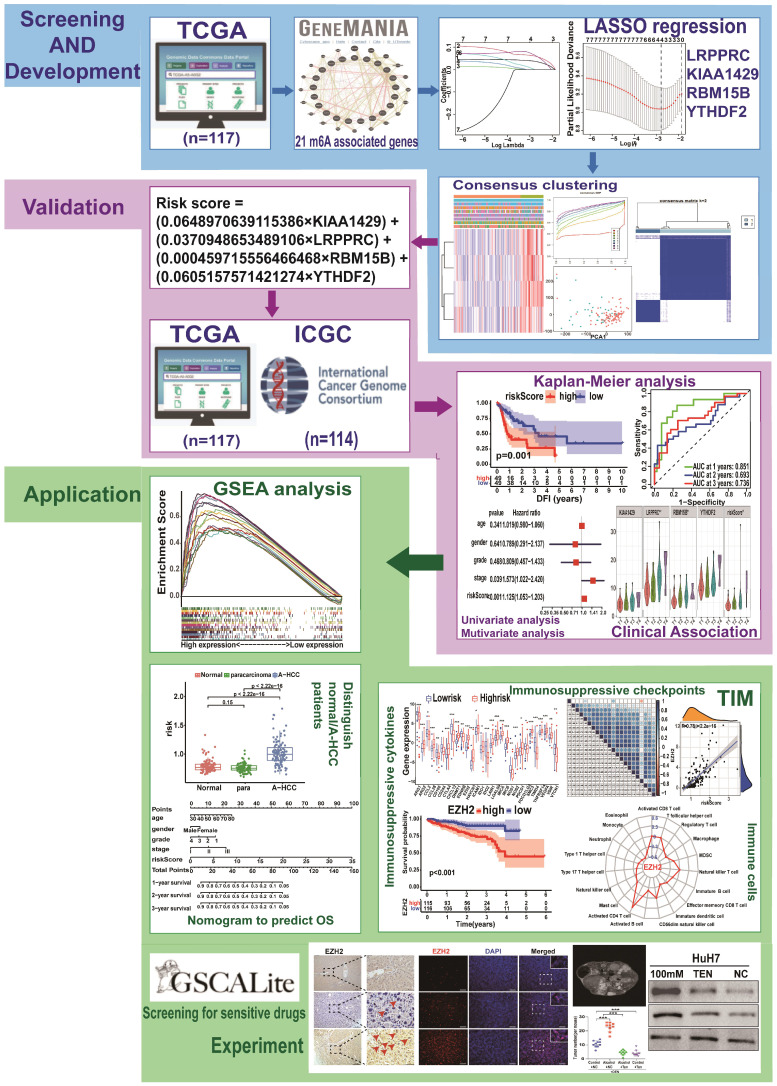
Flow chart of this study: establishment, verification, and application of m6A model.

**Figure 2:**
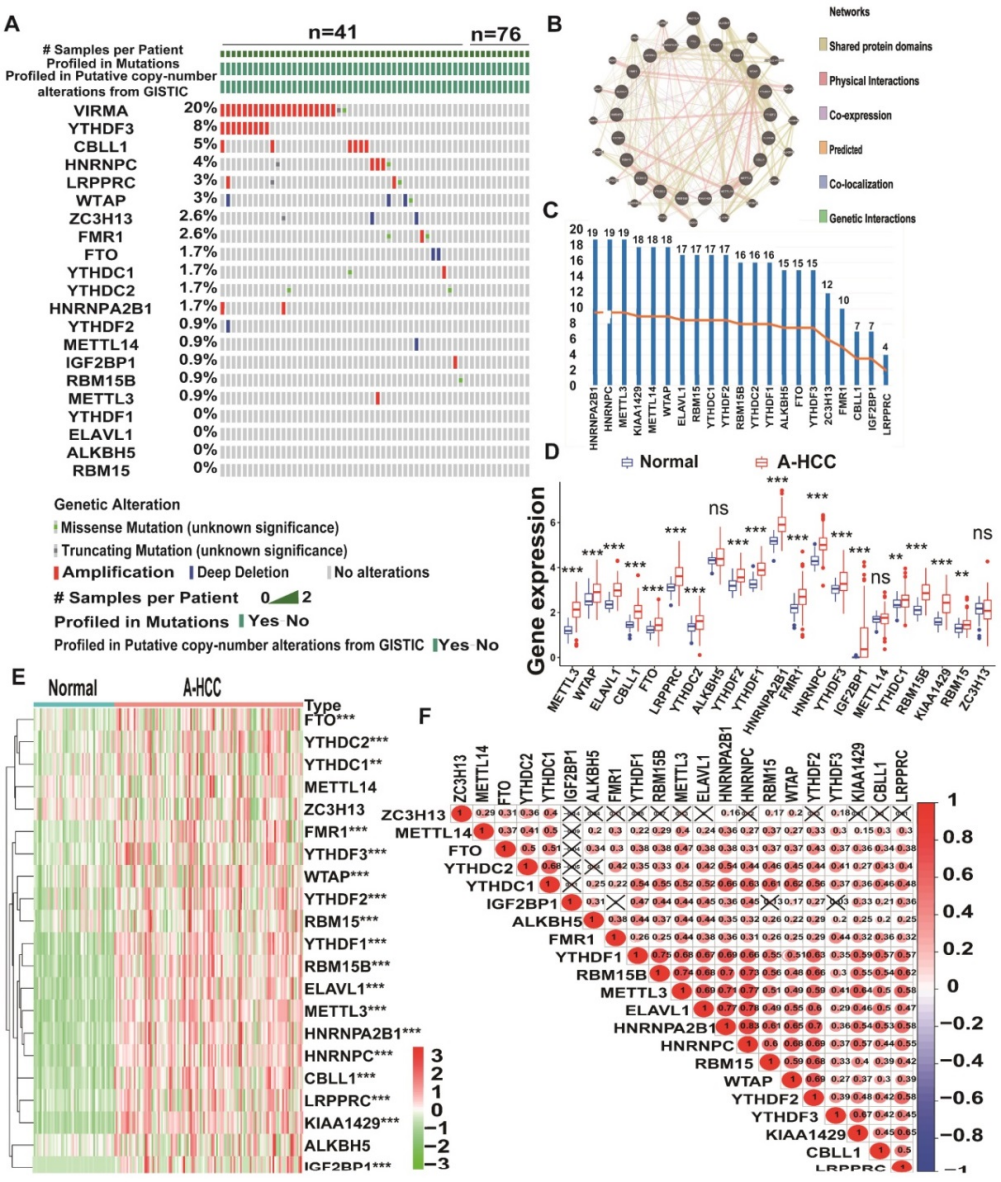
** Landscape of genetic expression and variation of m6A regulators in A-HCC. (A)** The mutation frequency of 21 m6A regulators in A-HCC patients from TCGA-LIHC cohort was acquired from Cbioportal. **(B)** Protein-Protein interactions among 21 m6A-related genes acquired from GeneMANIA. **(C)** Number of edges of 21 m6A regulators in the protein-protein interactions network. **(D-E)** Boxplot (D) and heatmap (E) of 21 m6A regulator expression levels between normal individuals and A-HCC patients. **(F)** Correlation analysis of 21 m6A regulators in TCGA-A-HCC cells.

The study design is shown in Figure 1. To determine whether the clinical prognosis of A-HCC is associated with known m6A-related genes, we summarised the occurrence of 21 m6A regulatory factor mutations in A-HCC in TCGA database (n = 117). Among them, *VIRMA* (KIAA1429) had the highest mutation rate (20%), followed by *YTHDF3*, whereas four genes (*YTHDF1, ELAVL1, ALKBH5*, and *RBM15*) did not show any mutation in this sample (Figure 2A). To systematically study all the functional interactions between proteins, we used the web site GeneMANIA to construct a network of interaction between the selected proteins and found that HNRNPA2B1 was the hub of the network (Figure 2B-C). Furthermore, we determined the difference in the expression levels of the 21 m6A regulatory factors between A-HCC and normal liver tissue (Figure 2D-E). Subsequently, we analysed the correlation of the m6A regulators (Figure 2F) and found that the expression patterns of m6A-regulatory factors were highly heterogeneous between normal and A-HCC samples, suggesting that the altered expression of m6A-regulatory factors might play an important role in the occurrence and development of A-HCC.

### An integrative m6A risk model

**Figure 3:**
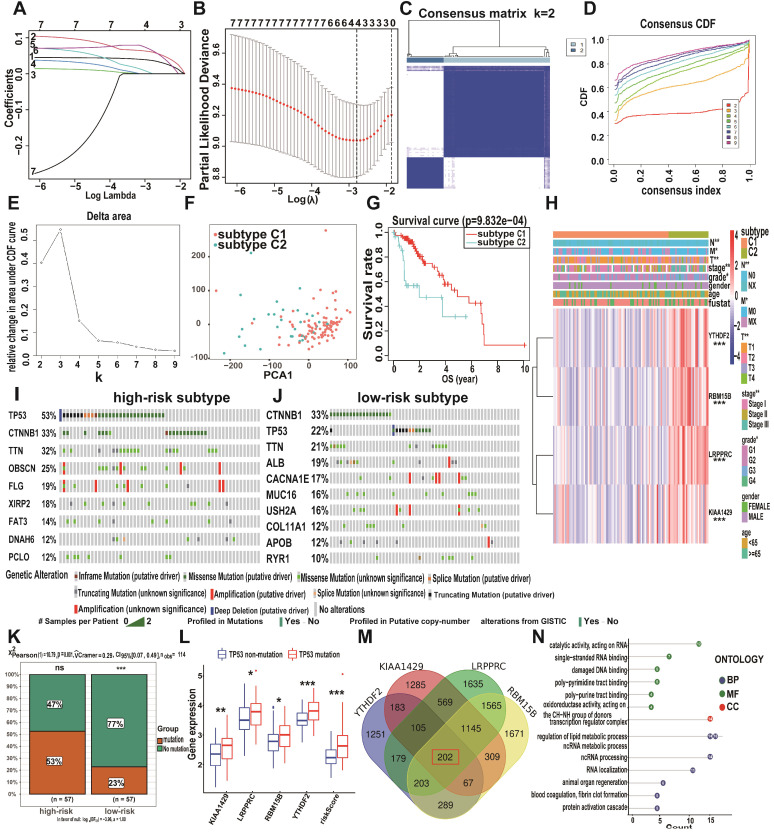
** Establishment of the model with four m6A RNA methylation regulators and availability of these key genes. (A-B)** Consensus clustering model with cumulative distribution function for k = 2-9 (k means cluster count). **(C)** TCGA A-HCC cohort was divided into two clusters when k = 2. **(D)** Relative change in area under cumulative distribution function curve for k = 2-9. **(E)** Consensus clustering cumulative distribution function for k = 2-9. **(F)** Principal component analysis of the total RNA expression profile in A-HCC cohort. **(G)** Overall survival curves for A-HCC patients. **(H)** Heatmap showing the relationship between two clusters and clinical characteristics. **(I & J)** m6A high/low risk subtype: the mutation frequency of the top 10 genes in different risk subtype with A-HCC from TCGA-LIHC cohort acquired from Cbioportal. **(K)** Chi-square test of mutation frequency in different risk subtypes. **(L)** Boxplots showing model-related gene expression and risk scores in the *TP53* mutation and non-mutation groups. **(M)** Venn diagram of *KIAA1429, LRPPRC, RBM15B,* and *YTHDF2* associated genes from Cbioportal. **(N)** GO analysis of *KIAA1429, LRPPRC, RBM15B,* and *YTHDF2* associated genes.

To explore the prognostic value of the expression levels of the 21 m6A methylation regulators in A-HCC, we performed univariate Cox regression analysis based on the expression levels of related factors in TCGA dataset and found seven related genes to be significantly related to OS (*p* < 0.05), namely *YTHDF2, KIAA1429, YTHDF1, RBM15B, LRPPRC, RBM15*, and *YTHDF3* (Supplementary Table 5). To identify the most powerful prognostic m6A regulator, we performed LASSO Cox regression analysis. Four candidate genes (*LRPPRC, KIAA1429, RBM15B,* and *YTHDF2*) were selected to construct the m6A risk assessment model (Figure 3A, B), the risk score was as follows: risk score = (0.0648970639115386 × *KIAA1429*) + (0.0370948653489106 × *LRPPRC*) + (0.000459715556466468 ×* RBM15B*) + (0.0605157571421274 × *YTHDF2).* Based on the expression levels of these four m6A-related genes as well as k = 2, a parameter that leads to Supplementary Table clustering outcome, we identified two new clusters in TCGA dataset (Figure 3C-E). Principal component analysis showed that cluster analysis could successfully divide A-HCC patients into two subtypes (Figure 3F). We compared the clinical survival curves of the two subtypes and found that the survival trend of subtype C1 was significantly better than that of subtype C2 (*p* = 9.832e-04; Supplementary Table 6, Figure 3G, Figure S1A). The expression levels of the four selected m6A-related genes and the clinicopathological variables in the two subtypes were closely related to tumour stage and grade (Figure 3H).

We verified the gene and protein expression of the four m6A regulators screened in the collected samples from HCC clinical patients, and the results showed that compared with normal patients, KIAA1429, LRPPCC, RBM15b and YTHDF2 were up-regulated in HCC patients, which was more significant in A-HCC patients (Figure S1B-C). Meanwhile, to further illustrate the external applicability of the model, we conducted survival analysis of the m6A model in a variety of cancers in addition to A-HCC and found that it was predictive (*p* =0.003), such as liver hepatocellular carcinoma (LIHC, *p* =0.01), lower grade glioma (LGG, *p* =0.029), uterine corpus endometrial carcinoma (UCEC,* p* =0.033) kidney chromophobe (KICH, *p* =0.005) and arenal cortical carcinoma (ACC, *p* =0.044; Figure S1D).

To further unravel the mutation events associated with the m6A risk model, we divided the A-HCC patients into high-risk and low-risk subtypes. In the high-risk subtype, 53% of the samples had mutations in *TP53* (Figure 3I), whereas *CTNNB1* mutations were frequent in the low-risk subtype (Figure 3J). *TP53* is a common tumour suppressor gene, and its mutations accompany tumorigenesis 34. The frequency of *TP53* mutations in the high-risk subtype was significantly higher than in the low-risk subtype (53% vs. 23%, *p* = 0.001; Figure 3K). Subsequently, we divided the A-HCC patients into two subtypes according to the presence or absence of mutations in *TP53* (Figure 3L). Risk scores and model-related gene expressions were higher in the *TP53*-mutation group than in the non-mutated group.

To explore the function of the four identified m6A-related genes, we extracted and screened genes their co-expressed genes and performed gene ontology (GO) enrichment analysis. A total of 202 genes were co-expressed with the four m6A-related genes (Figure 3M) and their functional categories were molecular function (MF), biological process (BP), and cellular component (CC). These terms were mainly enriched in pathways related to RNA processing, modification, and proliferation such as ncRNA metabolic processing and regulation of lipid metabolic processes (Figure 3N). Altogether, the results suggest that *TP53* mutation may be a key factor in initiating m6A methylation, which activates cancer-promoting pathways. Hence, the expression levels of *KIAA1429, LRPPRC, RBM15B,* and *YTHDF2* could be used as a prognostic indicator in A-HCC.

### Prognostic performance of the m6A risk model in A-HCC

**Figure 4:**
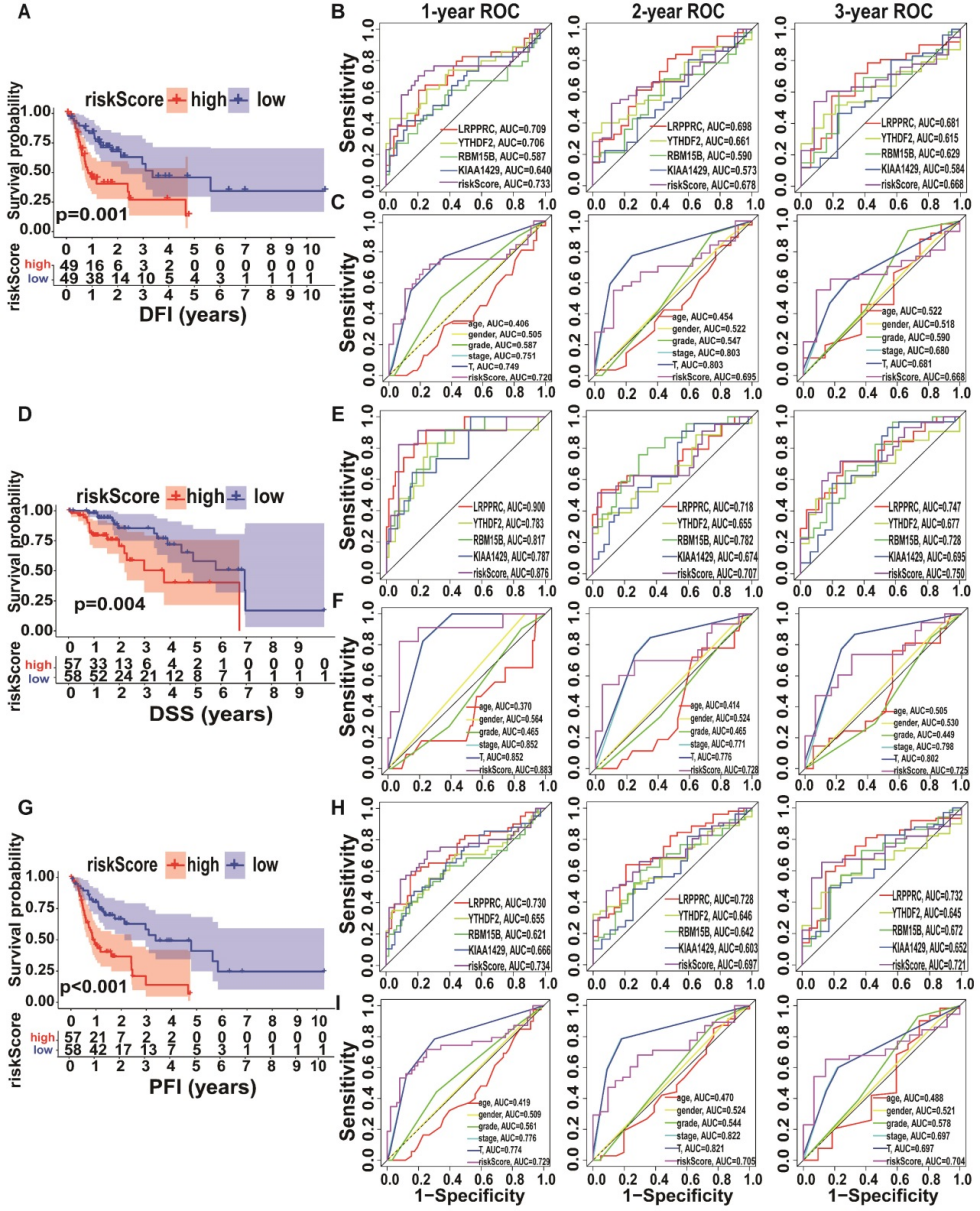
** Kaplan-Meier analysis and ROC curves of different survival times in the TCGA-A-HCC cohort. (A)** Different factors risk model of Kaplan-Meier analysis for disease-free interval (DFI). **(B-C)** Model-related genes (B)/ clinical characteristics (C) of ROC curves for DFI (1/2/3 year). **(D)** Different factors risk model of Kaplan-Meier analysis for disease-specific survival (DSS). **(E-F)** Model-related genes (E)/ clinical characteristics (F) of ROC curves for DSS (1/2/3 year). **(G)** Different factors risk scores of Kaplan-Meier analysis for progression-free survival (PFI). **(H-I)** Model-related genes (H)/ clinical characteristics (I) of ROC curves for PFI (1/2/3 year).

Since the expression levels of the four selected genes (*KIAA1429*, *LRPPRC*, *RBM15B*, and *YTHDF2*) play a crucial role in tumorigenesis and tumour development, we employed them to establish an m6A risk signature model. *KIAA1429*, *YTHDF2*, and *RBM15B* expression levels were not significantly different between the high/low-risk subtypes when DFI was analysed. In the LRPPRC low-expression group and low m6A risk model, patients had significantly longer DFI than those in the high-expression and high-risk model (Figure 4A, Figure S2A). To demonstrate the reliability of the m6A risk model, we constructed an ROC curve for DFI prediction and quantified the AUC. The AUC of the m6A risk model in 1/2/3 years was better than that of the expression of other genes (*KIAA1429*, *YTHDF2*, and *RBM15B*) and other factors, such as age, sex, and tumour grade (Figure 4B-C). The clinical prognostic differences were consistent from DSS and PFI analysis (Figure 4D-I), which indicated that the m6A risk model composed of four genes (*KIAA1429, LRPPRC, RBM15B,* and *YTHDF2*) can more accurately predict the prognosis of A-HCC.

**Figure 5:**
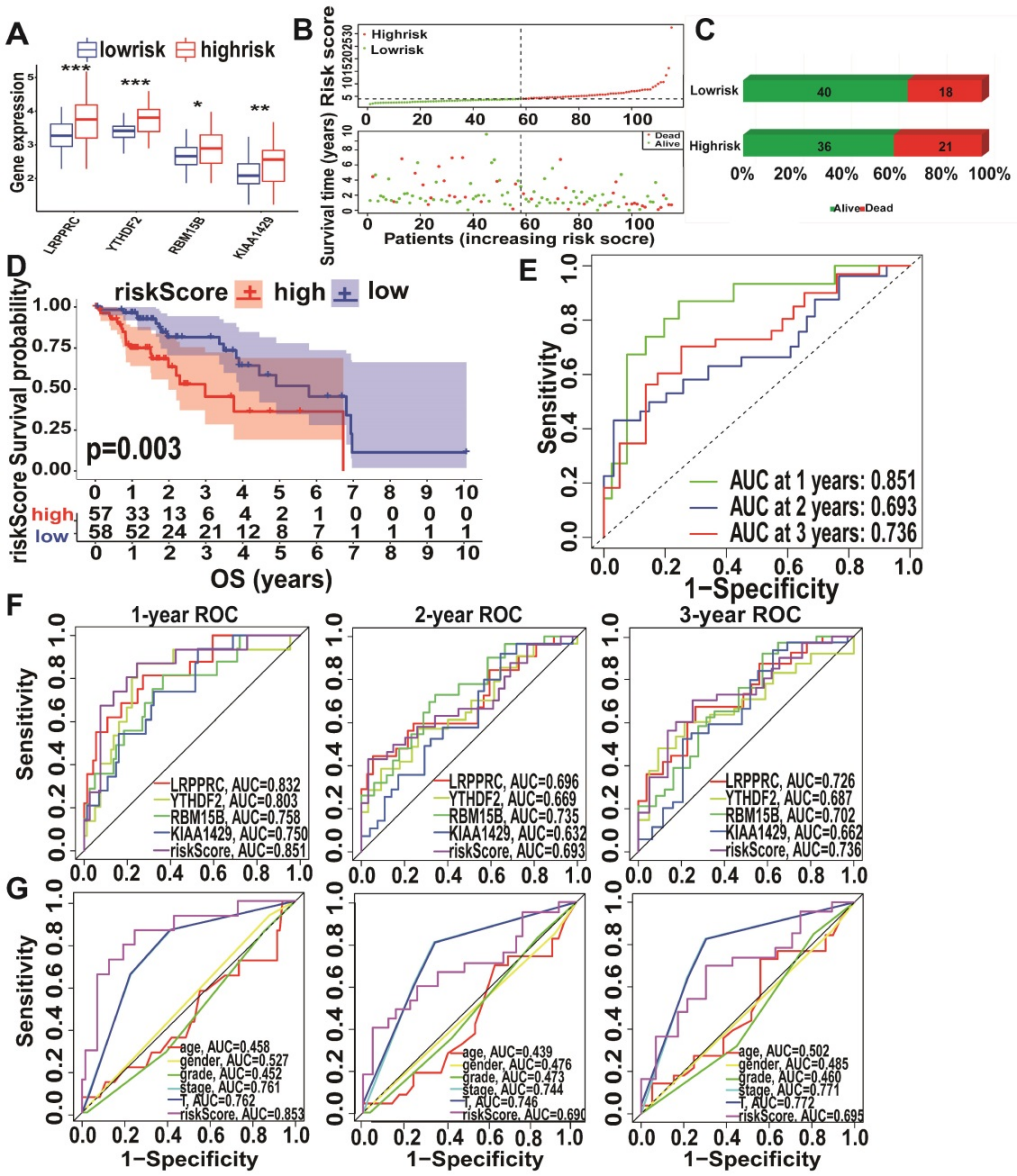
** Performance of the m6A-risk model in predicting A-HCC patient survival in TCGA databases. (A)** Boxplots showing four m6A-related gene expression profiles in high-risk and low-risk subtypes. **(B)** Patient status distribution in the high-risk and low-risk subtypes. **(C)** Mortality rates of the high-risk and low-risk subtypes. **(D)** Overall survival curves for A-HCC patients. **(E-G)** ROC curves of TCGA cohort: ROC curves showing the predictive accuracy of model (E)/model-related genes (F)/different clinical characteristics and time (1/2/3 year) (G).

Analysis of *KIAA1429*, *LRPPRC*, *RBM15B*, and *YTHDF2* expression levels in the high- and low-risk subtypes from TCGA database showed significantly upregulated expression in the high-risk subtype (Figure 5A). The high-risk subtypes had a lower OS and a higher risk score than those in the low-risk subtype (Figure 5B-C). Higher expression levels of *KIAA1429*, and *RBM15B* and higher m6A risk model scores were associated with a higher mortality rate in the high-risk subtype (Figure 5D, Figure S2D). To further evaluate the accuracy of the m6A risk model for predicting the 1, 2, and 3-year survival rate of A-HCC patients, we performed ROC curve analysis on TCGA (n = 117) cohorts (Figure 5E). Similarly, the performance of the m6A risk model was better than the models using the expression levels of a single gene and other factors (age, gender, tumour grade, tumour stage, and vascular invasion; Figure 5F-G). Meanwhile, the same verification was performed in the ICGC database (Figure S3). The above data show that the m6A risk model predicts the OS of A-HCC patients with more accuracy and reliability than any of the other models analysed.

### m6A risk model to evaluate the occurrence and development of A-HCC

**Figure 6:**
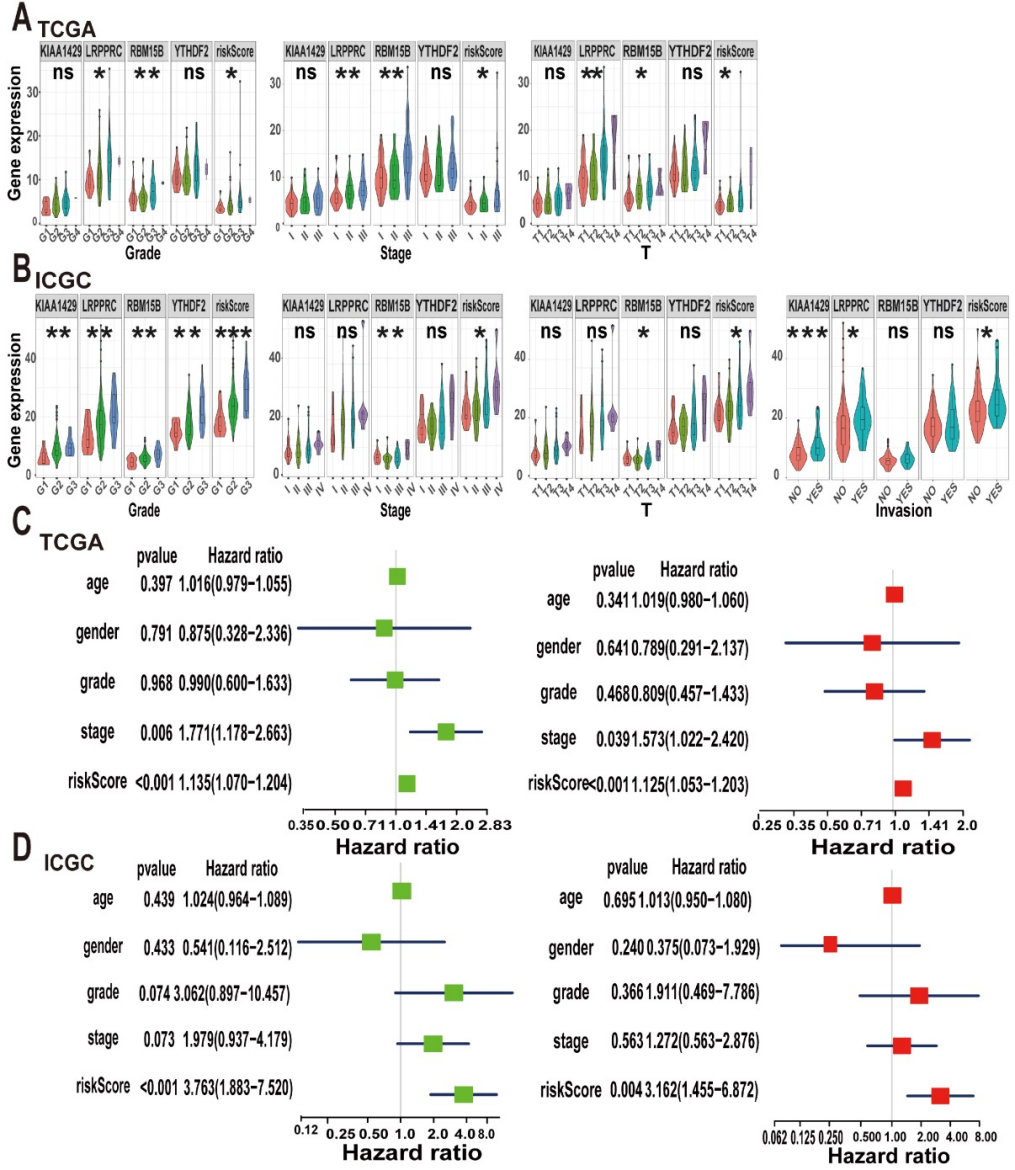
** Analysis of clinical characteristics analysis of the m6A-risk model in A-HCC. (A-B)** The expression levels of KIAA1429, LRPPRC, RBM15B, YTHDF2 and risk model in A-HCC patients with different clinical characteristics in TCGA (A) and ICGC (B) databases. **(C-D)** Univariate and Multivariate analyses in TCGA (C) and ICGC cohorts (D) in A-HCC patients; Left: Univariate evaluating m6A signature in terms of OS; Right: Multivariate analyses evaluating the m6A signature in terms of OS.

Considering that m6A methylation is closely related to the occurrence and development of tumours, we explored the relationship between the m6A risk model and clinicopathological characteristics. In TCGA cohort, the expression levels of *LRPPRC* and *RBM15B* and the m6A risk score were significantly correlated with tumour grade and T stage. Increases in tumour grade and T stage were associated with higher m6A risk score or gene expression levels. Additionally, *KIAA1429, LRPPRC,* and *RBM15B* and the m6A risk scores were significantly different between different tumour stages (Figure 6A). Subsequently, we validated our conclusions again using the ICGC dataset. In the ICGC cohort, *KIAA1429, LRPPRC, RBM15B,* and *YTHDF2* expression levels and the m6A risk score were significantly correlated with tumour grade. Moreover, the increase in tumour grade was associated with a gradual increase in the m6A risk model score. Only *RBM15B* expression levels and the m6A risk model score were associated with tumour stage and T stage. We also evaluated the relationship between the m6A risk model and vascular invasion and found that *KIAA1429* and* LRPPRC* expression levels and the m6A risk model score were significantly correlated with vascular invasion (Figure 6B). This indicates that tumour vascular invasion is highly correlated with the model score and that patients with higher scores are more likely to exhibit vascular invasion.

Next, we used the Cox regression model to perform univariate and multivariate survival analyses on the m6A risk model. In TCGA dataset, both univariate and multivariate analyses showed that tumour stage and the m6A risk model score were strongly associated with OS (Figure 6C), which was replicated in the ICGC database (Figure 6D). Thus, we concluded that the m6A risk model can used to evaluate the occurrence and development of A-HCC.

### GSEA signalling pathways

**Figure 7:**
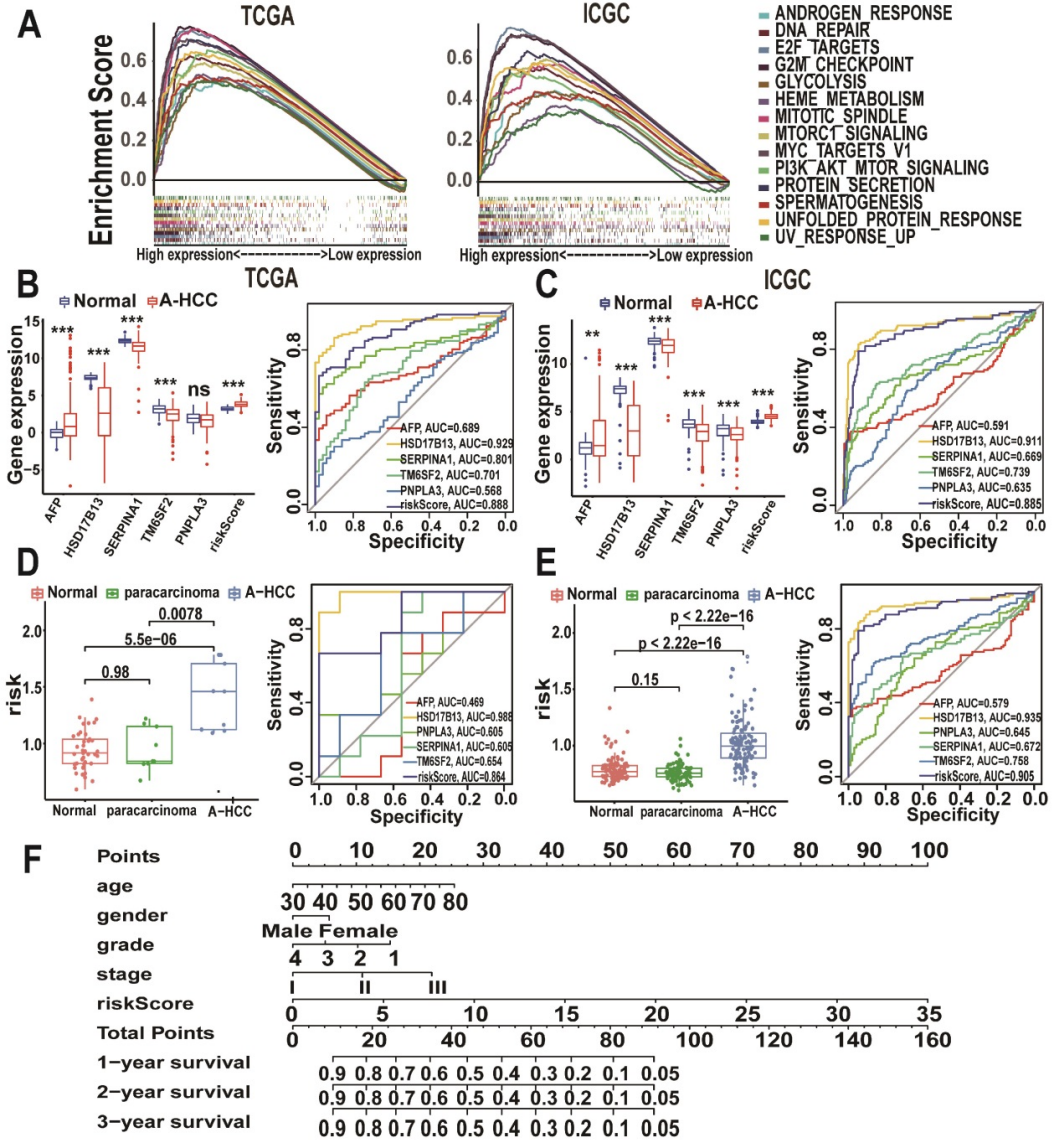
** Prognostic value of the m6A-risk model in A-HCC. (A)** GSEA showing enriched hallmarks in TCGA (left) and ICGC (right) cohorts. Normalized enrichment score (NES) > 1 and nominal p-value (NOM p-Val) < 0.05were indicated significant gene sets. **(B-C)** Boxplot and ROC curves (from left to right) of m6A-risk model in TCGA (B) and ICGC (C) cohorts to distinguish normal individuals and A-HCC patients. **(D-E)** Boxplot and ROC curves of the m6A-risk model in TCGA (D) and ICGC (E) cohorts to distinguish normal individuals and paracarcinoma and A-HCC patients. **(F)** Multivariate nomogram predicts OS in A-HCC patients.

To further explore the pathways potentially involved in the development of A-HCC, we divided the patients from TCGA and ICGC databases into high-risk and low-risk subtypes based on risk scores and performed GSEA enrichment analysis (Supplementary Table 7). Pathways enriched in the high-risk subtype were mainly related to tumour formation and proliferation, such as E2F targets, DNA repair, and MTORC1 signalling pathways (Figure 7A). Interestingly, the enriched pathways were shown to be closely related to tumour development and anti-apoptosis. For example, the E2F pathway plays a key role in cell proliferation by regulating the cell cycle 35.

### Utility of the m6A risk model in diagnosing and assessing the disease status of A-HCC

To explore the potential role of the m6A risk model in the diagnosis of A-HCC as well as its reliability and accuracy, we compared it with known A-HCC-related genes and diagnostic markers. Alpha-fetoprotein (AFP) is the most commonly used clinical HCC marker 36. Other proteins closely related to A-HCC include patatin-like phospholipase domain-containing protein 3 (PNPLA3), hydroxysteroid 17-beta dehydrogenase 13 (HSD17B13), serpin family A member 1 (SERPINA1), and transmembrane 6 superfamily member 2 (TM6SF2) 37-40. We found that the m6A risk model (AUC = 0.888) had a better predictive accuracy for A-HCC diagnosis compared with that of AFP, SERPINA1, TM6SF2, and PNPLA3 expression levels (Figure 7B). We validated these results using the ICGC database (Figure 7C).

We next evaluated the specificity of the m6A model in distinguishing A-HCC from alcohol-associated non-malignant changes. Surprisingly, the m6A risk model score was significantly increased in the A-HCC samples compared with A-HCC paracarcinoma and normal tissue samples in both TCGA and ICGC databases; additionally, the m6A model showed a marked sensitivity in A-HCC diagnosis (Figure 7D-E). We also verified that this model was superior to other related factors in distinguishing cancer and paracarcinoma tissue samples (Figure S4), demonstrating that dysregulated expression levels of m6A genes are highly specific in the tumorigenesis of A-HCC. Based on TCGA database, we established a nomogram to predict the OS of patients according to various possible influencing factors (Figure 7F).

### The immune landscape of A-HCC patients

**Figure 8:**
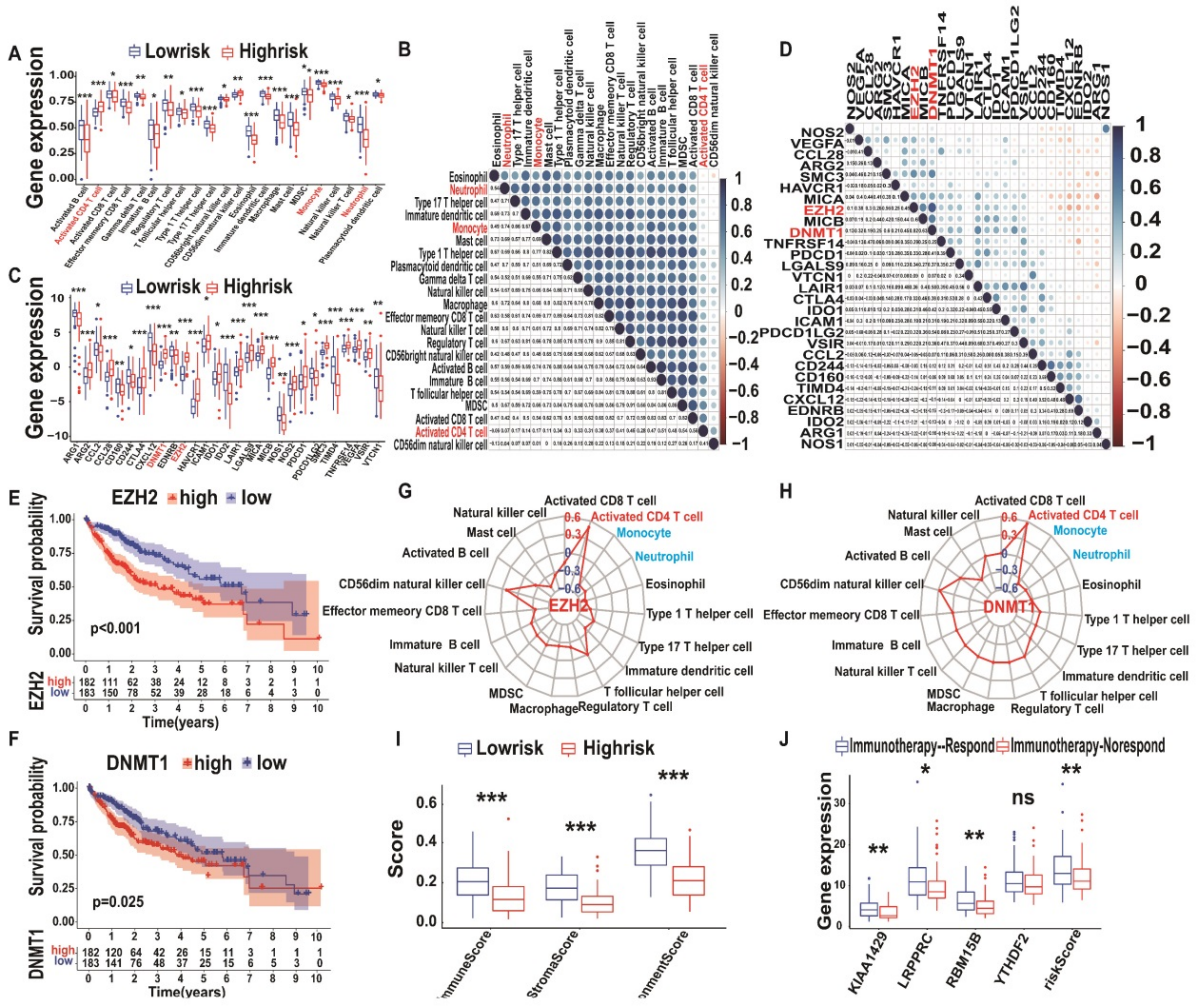
** Immune landscape and immunotherapy prediction between low and high m6A-risk A-HCC patients in TCGA databases. (A)** Boxplot visualizing the difference of immune cell infiltration among different risk subtypes from TCGA-A-HCC. * *P* < 0.05, ** *P* < 0.01, *** *P* < 0.001. **(B)** Correlation analysis of immune cells from TCGA-A-HCC. **(C)** Boxplot visualizing the different expression of immunosuppressive cytokines among different risk subtypes from TCGA-A-LIHC. * *P* < 0.05, ** *P* < 0.01, *** *P* < 0.001. **(D)** Correlation analysis of immunosuppressive cytokines from TCGA-A-HCC. **(E-F)** Kaplan-Meier analysis of DNMT1 (E) and EZH2 (F) for OS between different risk subtypes. **(G-H)** Radar map showing relationship between immune cells and DNMT1 (G)/EZH2 (H). **(I)** Boxplot of the relationship between ImmuneScore StromaScore ImmuneScore/StromaScore-MicroenvironmentScore. **(J)** Boxplot showing risk scores and four hub genes (KIAA1429, LRPPRC, RBM15B, and YTHDF2) between the immunotherapy non-response and immunotherapy response groups in the TCGA databases.

To explore the immune landscape of A-HCC patients, we performed ssGSEA using TCGA and ICGC databases. The resulting heatmap is shown in Figure S5A-B and the infiltration levels of various immune cell types are shown in Figure 8A. The infiltration levels of most immune-activated cells, such as activated CD8^+^ cells, activated CD8^+^ T cells, effector memory CD8^+^ T cells, gammadelta T cells, and immature B cells, were reduced in the high-risk subtype. However, the proportion of activated CD4^+^ T cells and CD56^dim^ natural killer cells in the high-risk subtype was higher than in the low-risk subtype. We then found a positive correlation between these immune cells, and the proportion of myeloid-derived suppressor cells were closely correlated with that of regulatory T cells (R = 0.91; Figure 8B). Subsequently, we downloaded the immunosuppressive cytokines related to the cancer-immunity cycle from the Tracking Tumour Immunophenotype website 41 and compared the relationship between the m6A risk model and immunosuppressive cytokines using box plots. The results showed that levels of most of the immunosuppressive cytokines, such as Arg2, CCL28, DNMT1, and EZH2, were upregulated in the high-risk subtype (Figure 8C), suggesting that high-risk A-HCC patients have reduced cancer-immunity cycle activity. Similarly, we analysed the correlation between these immunosuppressive cytokines and found that DNMT1 and EZH2 were highly correlated (R = 0.71; Figure 8D). Kaplan-Meier analysis of DNMT1 and EZH2 showed that patients with higher DNMT1/EZH2 expression have poorer OS (Figure 8E-F).

To verify the above conclusions, we generated a Venn diagram of immune cells and immunosuppressive cytokines of TCGA/ICGC databases, which resulted in an overlap of 19 immune cells and 26 immunosuppressive cytokines (Figure S4C). Subsequently, we explored the correlation between immunosuppressive cytokines (DNMT1 and EZH2) and all immune cells (Supplementary Table 8) and found that three types of immune cells (activated CD4^+^ T cells, monocytes, and neutrophils) were closely related to DNMT1 and EZH2 levels (Figure 8G-H). These results were further validated in the ICGC database (Figure S6A-H). Therefore, we performed logistic regression analysis of the model risk score and immune cells/immunosuppressive cytokines levels and found that they were closely correlated (Figure S7). Altogether, these results indicate that an increase in activated CD4^+^ T cell infiltration is associated with higher expression levels of DNMT1 and EZH2, whereas the opposite was observed for monocyte and neutrophil infiltration. Therefore, immunosuppressive cytokines, such as DNMT1 and EZH2, and immune cells, such as activated CD4^+^ T cells, monocytes, and neutrophils, may form a TIM regulatory system, representing a new target for A-HCC therapy.

### m6A model predicts A-HCC treatment efficacy

In TCGA database, patients in the m6A high-risk subtype had lower immune and stroma scores as well as lower ration immune score - stroma Score/microenvironment score than patients in the m6A low-risk subtype (Figure 8I). Thus, our model could predict the TIM state and the therapeutic responses of A-HCC. Recently, an ImmuCellAI estimation method was developed to predict the response of HCC patients to immunotherapy 42. We evaluated whether the m6A risk model can make similar predictions and analysed the difference in *KIAA1429*,* LRPPRC*,* RBM15B,* and *YTHDF2* expression levels and the risk score between the responder and non-responder group.

Significantly upregulated expression of *KIAA1429*, *LRPPRC*, and *RBM15B* and high-risk scores were observed in the non-responder group compared with the responder group (Figure 8J). This was further verified using the ICGC database (Figure S5I-J). As shown in Figure 8A, most high-risk subtypes lacked immune cells; immunoreactive cell deficiency is known to cause immunotherapy tolerance 43, 44, which indicates that high-risk may be related to non-response to immunotherapy (immune tolerance).

We further conducted a drug sensitivity analysis of DNMT1, EZH2, RBM15B, KIAA1429, LRPPRC, and YTHDF2 using the CTRP database. Screening revealed teniposide, PX-12, LRRK2-IN-1, and GSKJ4 as potential therapies for A-HCC (Figure S8).

### Validation of A-HCC core genes (DNMT1/EZH2) and potential drugs

**Figure 9:**
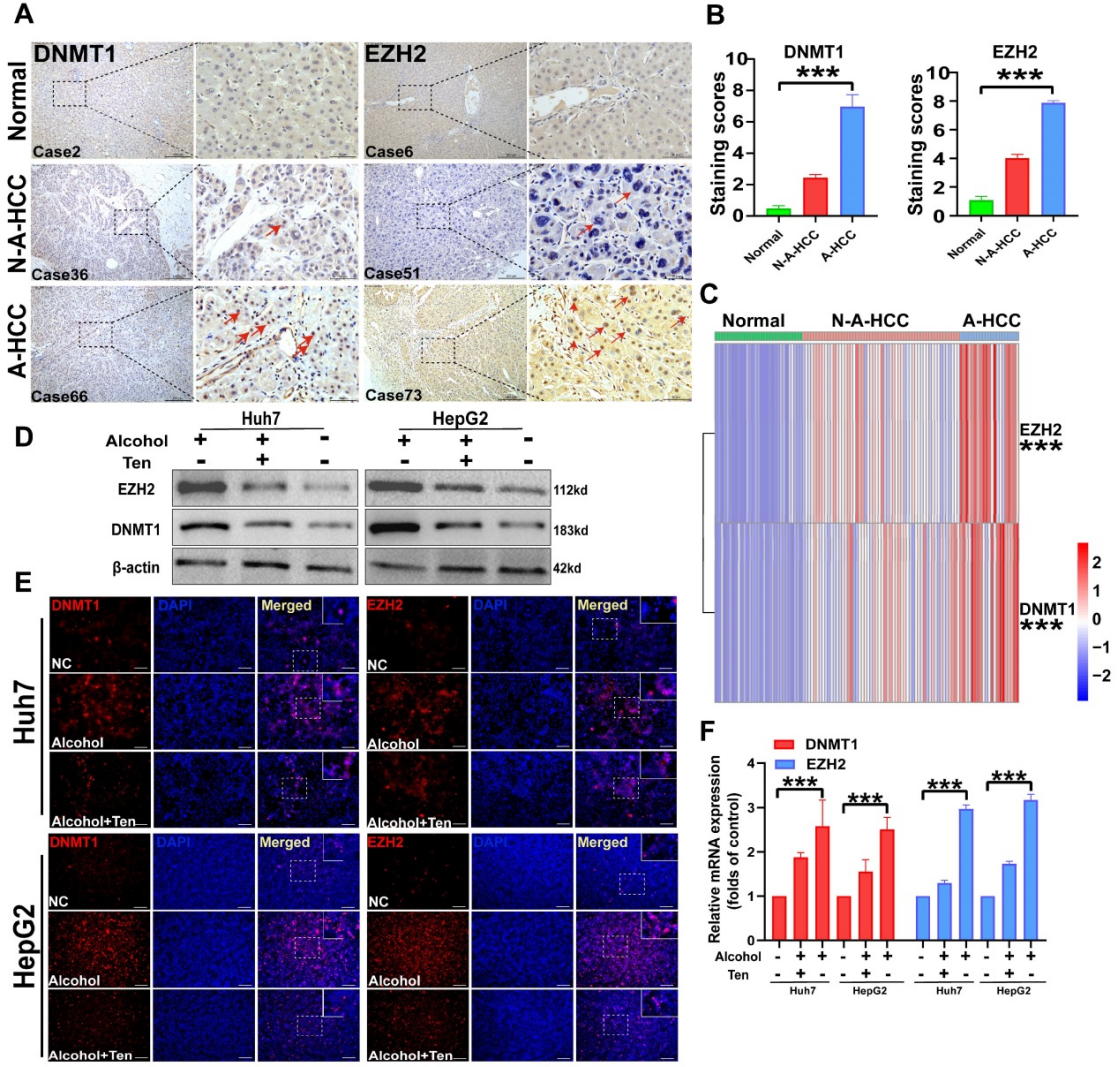
** Expression of DNMT1/EZH2 and potential drug validation. (A-B)** The immunohistochemical staining of DNMT1/EZH2 in clinical patients of three groups was observed: Normal (n = 31), N-A-HCC (no history of alcohol consumption n = 56), and A-HCC (n = 21) (A), the positive rate of immunohistochemical staining was analysed (B). **(C)** qRT-PCR expression of DNMT1/EZH2 in clinical patients of the three groups (Normal/ N-A-HCC/ A-HCC). **(D-F)** HCC cell lines (Huh7 and HepG2) were treated with alcohol, divided into normal control (NC) group, alcohol (100 mM) groups, and teniposide group (0.5 μM teniposide treatment of alcohol-treated HCC cells), and the expression of DNMT1/EZH2 was analysed using western blotting (D), immunofluorescence staining (E) and qRT-PCR (F).

We collected pathological samples from normal, N-A-HCC, and A-HCC patients and performed immunohistochemical staining and qRT-PCR. DNMT1 and EZH2 levels in the liver tissues of normal individuals and N-A-HCC patients were barely detecSupplementary Table, while they were diffusely expressed in A-HCC patients (Figure 9A-C), indicating that DNMT1 and EZH2 expression in A-HCC patients is increased in comparison with normal and N-A-HCC individuals.

We then evaluated the role of DNMT1 and EZH2 in guiding A-HCC treatment. As the therapeutic effects of PX-12 45, LRRK2-IN-1 46, and GSK-J4 47 in A-HCC have been already described, we decided to explore the therapeutic effect of teniposide on A-HCC. We employed two HCC cell lines, Huh7 and HepG2, and treated them with 100 mM alcohol, as a cellular model of A-HCC. DNMT1 and EZH2 gene expression and protein levels, evaluated by qRT-PCR, western blotting and immunofluorescence staining, were significantly higher in the alcohol-treated group (100 mM) than in the control group. Administration of teniposide (0.5 μM) to alcohol-treated cells abolished these effects (Figure 9D-F). Given that DNMT1 and EZH2 are barely expressed in the control group but are significantly up-regulated by alcohol-treatment and significantly down-regulated after teniposide treatment, the results suggest that DNMT1 and EZH2 may be core proteins in the aetiology of A-HCC and highlight teniposide as a potential therapeutic drug.

### The potential therapeutic effect of teniposide against A-HCC *in vivo*

**Figure 10:**
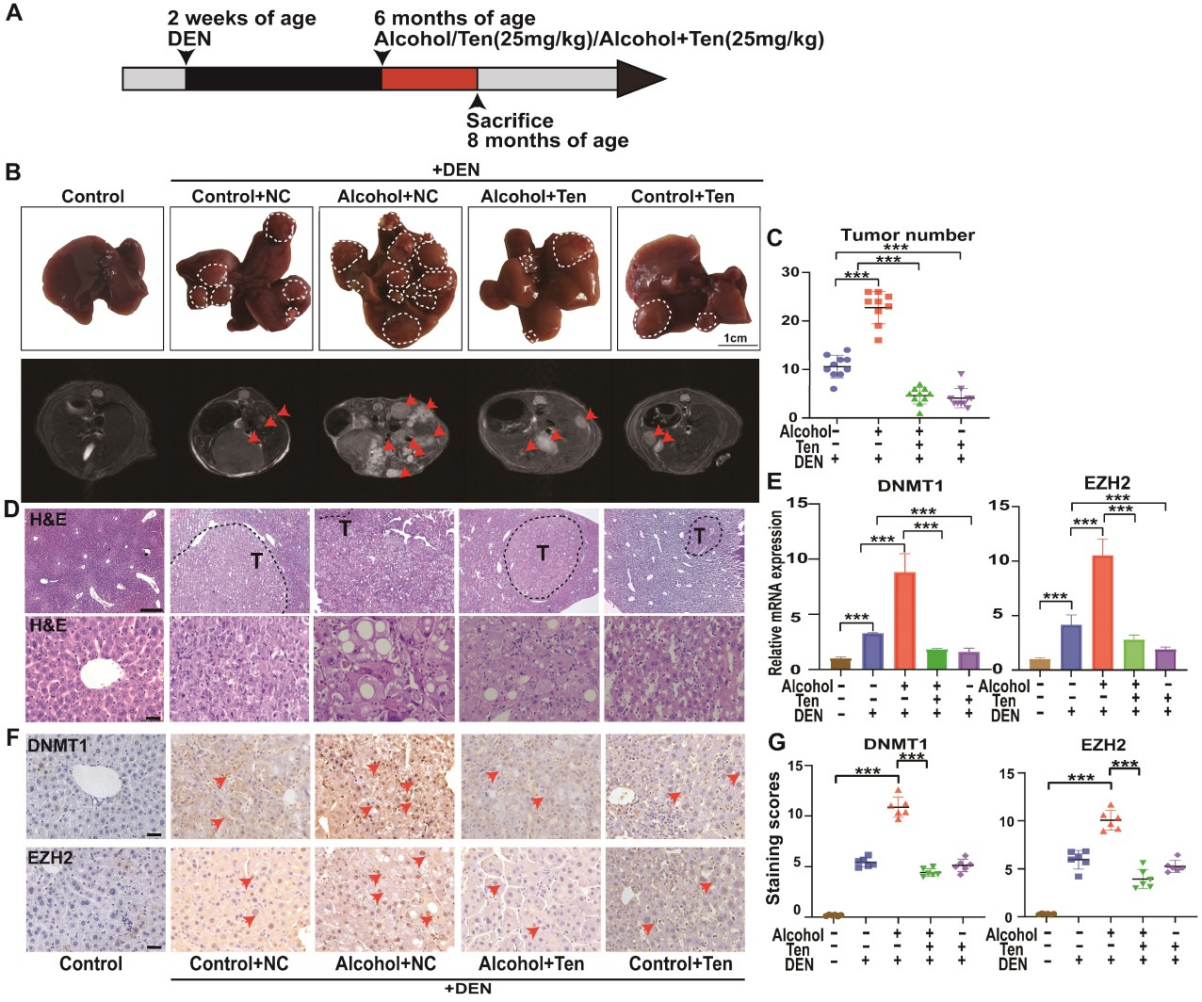
** Alcohol promotes the occurrence and development of HCC, while teniposide showed a therapeutic effect on A-HCC. (A)** C57BL/6 mice were injected with 25mg/kg DEN (n = 40) at 2 weeks of age. At 6 months of age, mice were given liquid alcohol diet (4.8% alcohol) (n = 10), teniposide (n = 10) and liquid alcohol diet (4.8% alcohol) + teniposide (n = 10). The animals were sacrificed at 8 months of age. **(B)** Representative MRI liver images obtained for each group (arrows depict tumours), n = 10 for Control without DEN stress, Control+NC, Alcohol+NC, Alcohol+Ten, and Control+Ten group under DEN stress. **(C)** Quantification results of tumour number for each group under DEN stress, *** *P* < 0.001 (Mann-Whitney *U* test). **(D)** H&E staining of representative liver sections observed in each group (dotted line indicates tumours outline). Scale bar, 400 µm (D); 50 µm (E). **(E)** qPCR results of DNMT1 and EZH2 in liver tissue of each group, data shown as mean ± SEM; n = 10 for Control without DEN stress, Control+NC, Alcohol+NC, Alcohol+Ten, and Control+Ten group under DEN stress, *** *P* < 0.001 (Mann-Whitney *U* test). **(F-G)** Immunohistochemical staining showed the expression level of DNMT1/EZH2 in each group: Control without DEN stress, Control+NC, Alcohol+NC, Alcohol+ TEN, and Control+ TEN group under DEN stress (F), positive rate of immunohistochemical staining was analysed. Scale bar, 40x, 50 µm. *** *P* <0.001 (Mann-Whitney *U* test) (G).

We evaluated the role of teniposide in the occurrence and development of HCC in mice; an overview of the experimental procedure is provided in Figure 10A. Mice begin to form HCC 7-10 months after injection of DEN solvent 48; hence we administered alcohol and drugs (teniposide, TEN) at 6 months of age, and divided the mice into five groups: Control+NC (without TEN and alcohol), Alcohol+NC (without TEN), Alcohol+TEN, Control+TEN (without alcohol) under DEN stress, and Control without DEN stress (10 mice per group). MRI imaging (AVANCE IIITM HD 600MHz) of the mouse liver was obtained at 8 months and representative liver images of each group are shown in Figure 10B. Tumour number analysis showed that teniposide significantly reduced tumours numbers in A-HCC (Figure 10C). Haematoxylin and eosin staining of liver sections demonstrated that Alcohol+NC group had the most obvious liver lesions and that teniposide was more effective in treating A-HCC than HCC (Figure 10D). We then determined the expression of two A-HCC core genes (*DNMT1* and *EZH2*) using qRT-PCR and IHC, and found significantly higher expression in the A-HCC group than in the HCC group, which significantly decreased following teniposide treatment (Figure 10E-G). Taken together, these data suggest that teniposide has a potential therapeutic effect on the occurrence and progression of A-HCC by acting on the A-HCC core genes, *DNMT1* and *EZH2*.

## Discussion

**Figure 11:**
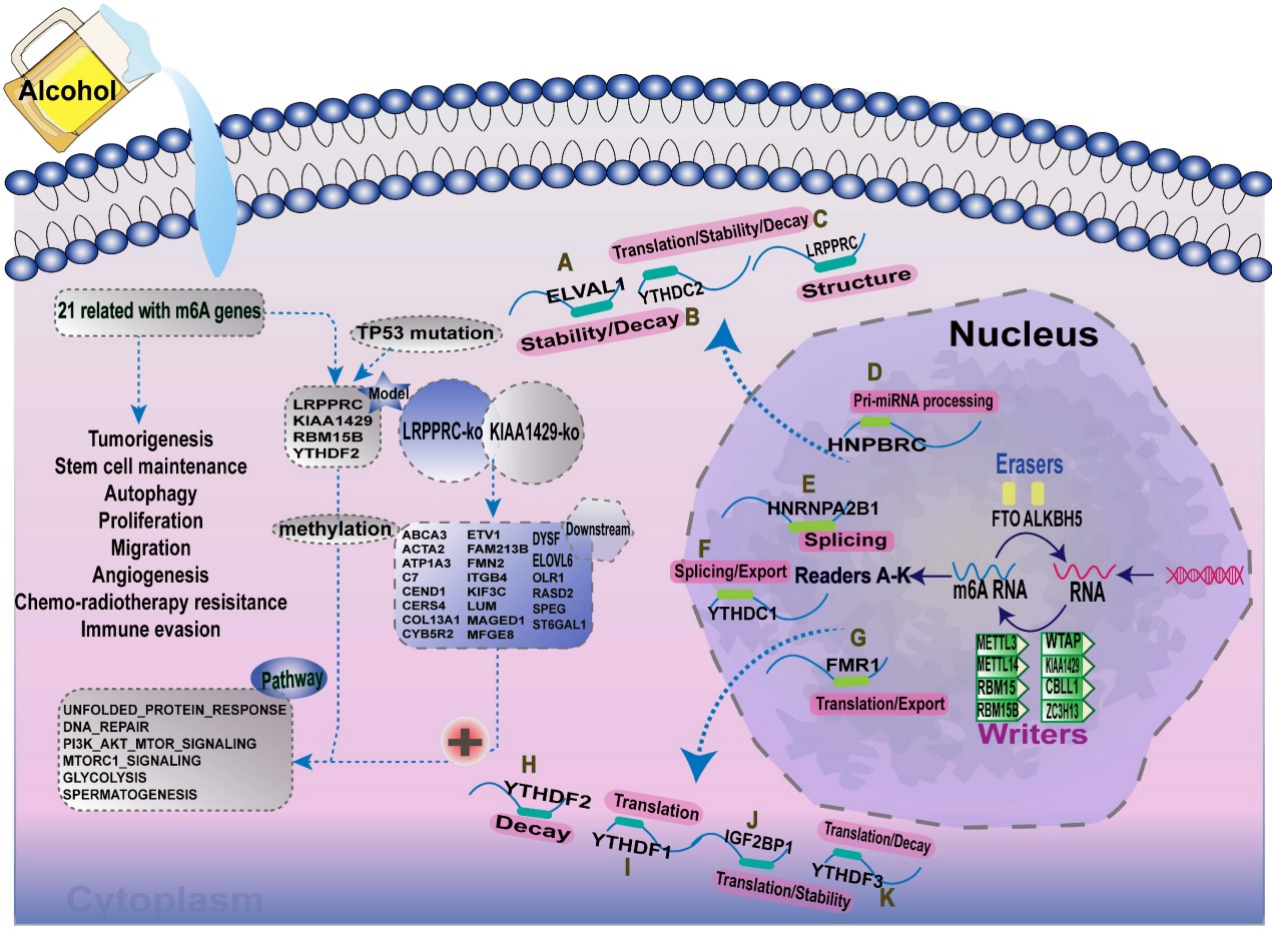
Summary of the dynamic process of m6A RNA methylation mediated by 21 regulators and their potential biological functions.

Increasing evidence has demonstrated that the interaction between multiple m6A regulators plays an essential role in the development and progression of several types of cancers. Here, we summarised the m6A-regulatory genes involved in the pathways associated with tumorigenesis (Supplementary Table 9). To clarify the relationship between m6A-related genes and the prognoses of patients with A-HCC, we selected 21 m6A regulators and mapped the m6A modifications mediated by these regulators and their potential biological functions in disease occurrence (Figure 11). Demethylases (FTO and ALKBH5) and methyltransferases (such as Metl3 and Metl14) have been reported to regulate the progression of several types of cancers, including liver, lung, and breast cancers 49-52. For example, silent information regulator 1 (SIRT1) can deregulate FTO to guide the m6A methylation of downstream molecules 53, and ALKBH5 can act as a tumour suppressor by reducing the expression of LYPD1 in HCC 54.

Currently, the mortality rate of HCC remains high, especially for A-HCC patients, owing to the lack of early diagnostic markers, treatment resistance, and poor immunotherapy sensitivity 55. The TIM plays a decisive role in the effectivity of treatment methods and can lead to tumour development and recurrence after treatment 56, 57. In the progression of liver cancer, immune cells and immunosuppressive cytokines can adjust the balance of the immune system by, for example, modifying the proportion of T-cell subsets 58. Importantly, the immunosuppressive state in the tumour can be transformed into an immune-activated state by adjusting the TIM and inducing an effective immune response, which can lead to an enhanced sensitivity to immunotherapy. Therefore, evaluating the TIM, and its relationship with prognosis in A-HCC patients is urgent. In the present study, we clarified the role of m6A methylation in regulating the TIM and developed a risk model based on the expression levels of m6A regulators, which may help predict the prognosis of A-HCC patients and more effective immunotherapy targets.

The risk model developed herein integrated four m6A methylation regulators, *LRPPRC, YTHDF2, KIAA14219,* and* RBM15B*. *LRPPRC* encodes a nuclear protein that is significantly negatively correlated with the immune response in a variety of tumour tissues 59-61. *YTHDF2* encodes a member of the YT521-B homology (YTH) domain family that has been reported to regulate mRNA stability 62, 63, and Yu et al. found that *YTHDF2* plays a negative regulatory role in the inflammatory response induced by LPS 64. *KIAA14219* encodes a methyltransferase that acts as a m6A 'writer' 65 and has been reported to be involved in tumour cell proliferation and metabolism pathways in liver and breast cancers 66, 67. Finally, *RBM15B*, which encodes another m6A 'writer', modifies m6A by binding to m6A methylation complexes (METTL3 and WTAP) 68. *RBM15B* has been reported to be associated with the immune landscape in various diseases 69.

In this study, we used the four m6A regulators to divide A-HCC patients into two subtypes and predicted their prognosis, and the model was validated in clinical patient samples we collected. We notably found that m6A high-risk subtypes had a high frequency of mutations in *TP53*. As *TP53* is a tumour suppressor gene, this indicates that* TP53* mutations may cause changes in m6A methylation levels. In addition, the pathways associated with the high-risk subtype were mainly related to RNA processing modification, and tumour development, suggesting that these four m6A regulators can be used as indicators of the occurrence and prognosis of A-HCC. In analysing different survival interval (DFI, DSS, PFI and OS), we found that the prognosis of the m6A high-risk subtype was significantly worse and that the m6A risk model was more reliable and accurate than single genes in prediction efficiency, which could be used as an independent predictor. Meanwhile, the model was more reliable than the common clinical indicators AFP, PNPLA3, HSD17B13, SERPINA1, and TM6SF2 in predicting patient outcome. Finally, we constructed a nomogram based on various confounding factors, with the aim of applying this model to clinical guidance in the future.

GSEA indicated that the pathways enriched in the high-risk subtype were related to tumour formation and proliferation, which included the common E2F pathway and the PI3K/Akt/mTOR pathway 70, 71. E2F is a transcription factor that controls the expression of all cell division genes, of which E2F8 is significantly increased in HCC and ovarian cancer 72. It can transcriptionally inhibit CDK1-induced hepatocyte polyploidy, interact with HIF1 to form a complex, improve VEGFA level, promote angiogenesis, and induce tumour metastasis 72, 73. In addition, the PI3K/Akt/mTOR pathway is essential for tumour survival and growth, and induces resistance to radio-therapy, chemo-therapy, and cytostatic drugs 74.

A large amount of data from various disease conditions have indicated a correlation between m6A modifications and TIM 75-77. Although several studies have investigated the role of single regulatory factors or a single immune-infiltrating cell type in the immune response 78, 79, the comprehensive role of multiple m6A regulators in the immune response has not been studied to date. In this study, we describe the relationship between m6A regulators and the A-HCC immune response. In our model, there were clear differences in the TIM cell infiltration characteristics, higher m6A risk scores were associated with a higher infiltration of activated CD4^+^ T cells, higher levels of immunosuppressive cytokines (DNMT1 and EZH2) and reduced levels of monocytes and neutrophils infiltration. These features indicate an immunosuppressive TIM in the high-risk subtype, corresponding to the so-called 'immune desert type'. In contrast, the low-risk subtype had an immune-activated state. Therefore, the immunosuppressive cytokines DNMT1 and EZH2; and the immune cells activated CD4^+^ T cells, monocytes, and neutrophils appear to form a TIM regulatory system that greatly impacts the prognosis of A-HCC.

DNMT1, a common DNA methyltransferase, is involved in DNA methylation in eukaryotes 80. DNMT1 is closely related to the occurrence and development of various diseases, including several types of cancers, as it affects the methylation levels of CD4+T cell-related genes, thereby inhibiting the immune response 81-84. EZH2 acts as a catalyst for polycomb repressive complex 2 (PRC2) formation, catalysing the trimethylation of lysine 27 on histone H3 (H3K27me3) and mediating gene silencing 85. Several studies have reported that EZH2 can regulate the development and function of B cells and neutrophil migration and change the plasticity of CD4+T cells, highlighting the important role of EZH2 in the immune regulation of many diseases 86-88.

CD4^+^ T cells act as central orchestrators of immune regulation. Depending on the specific TIM, activated CD4^+^ T cells can differentiate into CD4+ T helper (Th) cells, which collaborate with B cells and CD8+ T cells promote immune response 89, 90. Monocytes are an important part of innate immunity and have been reported to be key regulators of cancer development 91. During tumorigenesis, monocytes perform several antitumor immunity functions, including phagocytosis and recruitment of lymphocytes, and can even differentiate into tumour-related immune cells 92, 93. Neutrophils exhibit powerful antimicrobial functions, including phagocytosis and formation of neutrophil extracellular traps 94, 95. Under pathological conditions, neutrophils are activated and infiltrate lesions, thereby changing the tissue microenvironment 96-98.

We evaluated the performance of the m6A risk model in assessing the sensitivity of immunotherapy and found that high score models were associated with reduced sensitivity to treatment. This may be because activated CD4^+^ T cells, monocytes, and neutrophils in the m6A high-risk subtype interact with DNMT1 and EZH2, resulting in an immunosuppressive, desert type microenvironment. DNMT1 and EZH2 expression levels were then compared between normal, N-A-HCC and A-HCC samples, while activating activated CD4T cells and inhibiting monocyte and neutrophil. DNMT1 and EZH2 expression levels were revealed to be correlated with changes in immune cells in the TIM and may improve the TIM state by inhibiting its expression. Through drug sensitivity analysis, we found that A-HCC patients were generally sensitive to teniposide, PX-12, LRRK2-IN-1, and GSK-J4 drugs, which can help clinicians better select treatment strategies. Among these four drugs, teniposide has not been reported in HCC studies. In our study, we found that teniposide has a potential therapeutic effect on A-HCC by down-regulating the expression of A-HCC core genes (DNMT1 and EZH2), thereby reversing the malignant degree of A-HCC and improving the prognosis.

In conclusion, we employed the expression levels of m6A regulators to construct a risk model that can accurately predict the prognosis of A-HCC patients and aid further understanding of the TIM state in A-HCC. The model can also predict the sensitivity of A-HCC patients to immunotherapy and drug therapy, which can greatly help guide future clinical selection of A-HCC targeted therapy and immunotherapy. Our finding also demonstrated that DNMT1 and EZH2 can be exploited as core genes of A-HCC and that teniposide can be used for the treatment of A-HCC.

## Supplementary Material

Supplementary figures and tables.

## Abbreviations

A-HCC: alcohol-induced HCC
AUC: area under the curve
DFI: disease-free interval
DMEM: Dulbecco's modified Eagle's medium
DSS: disease-specific survival
FBS: foetal bovine serum
HCC: hepatocellular carcinoma
ICGC: International Cancer Genome Consortium
LASSO: least absolute shrinkage and selection operator
LPS: lipopolysaccharide
OS: overall survival
PFI: progression-free interval
ROC: receiver operating characteristic
TCGA: The Cancer Genome Atlas
TIM: tumour immune microenvironment
LGG: lower grade glioma
UCEC: uterine corpus endometrial carcinoma
KICH: kidney chromophobe
ACC: arenal cortical carcinoma
