# Identification and Characterization of Alcohol-related Hepatocellular Carcinoma Prognostic Subtypes based on an Integrative N6-methyladenosine methylation Model: Erratum

**DOI:** 10.7150/ijbs.79940

**Published:** 2022-11-03

**Authors:** Yue Zhang, Fanhong Zeng, Min Zeng, Xu Han, Lei Cai, Jiajun Zhang, Jun Weng, Yi Gao

**Affiliations:** 1 Department of Hepatobiliary Surgery II, Guangdong Provincial Research Center for Artificial Organ and Tissue Engineering, Guangzhou Clinical Research and Transformation Center for Artificial Liver, Institute of Regenerative Medicine, ZhuJiang Hospital, Southern Medical University, Guangzhou, Guangdong Province, China.; 2 State Key Laboratory of Organ Failure Research, Southern Medical University, Guangzhou, China.

In our paper, the author noticed an error in Figure 7F. There is a non-subjective typographical error in the multivariate nomogram (Figure 7F). We checked the original data again and made sure that the conclusion of the article was not affected by the error. In this regard, all authors have agreed to the erratum, and we apologize for any inconvenience caused by the negligence in our work.

Figure 7F should be corrected as follows.

## Figures and Tables

**Figure 7:**
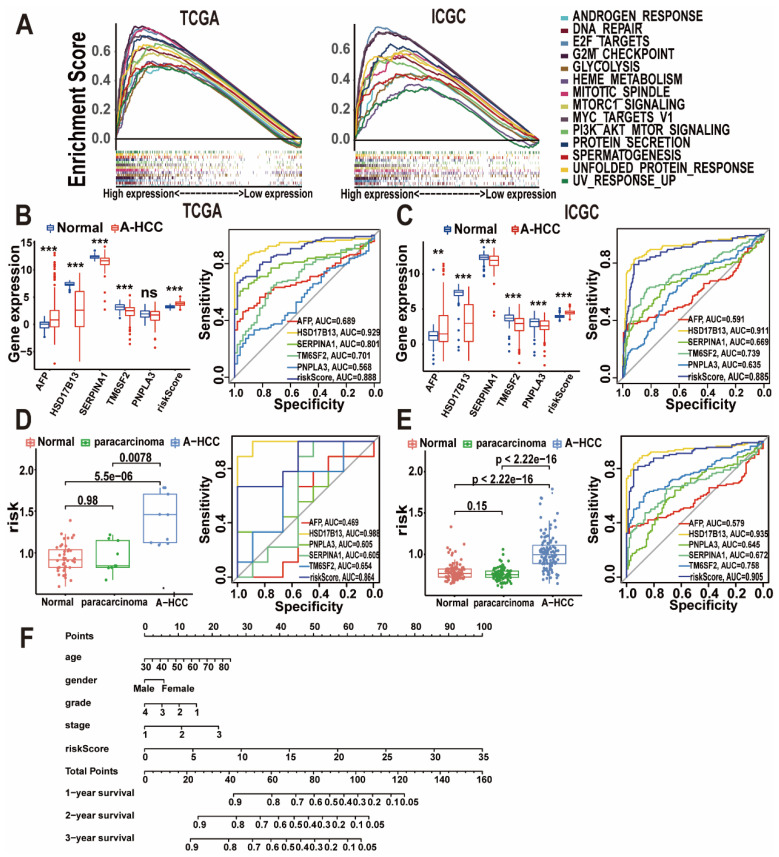
Correct image.

