# Supplementary material for: Identification and Characterization of Alcohol-related Hepatocellular Carcinoma Prognostic Subtypes based on an Integrative N6-methyladenosine methylation Model
Source: Int J Biol Sci. 2021 Aug 14;17(13):3554–72. doi: 10.7150/ijbs.62168 (PMC8416726; doi:10.7150/ijbs.62168)
Supplement: Supplementary file 1 — Supplementary figures and tables. [file ijbsv17p3554s1.pdf]

Supplementary Material

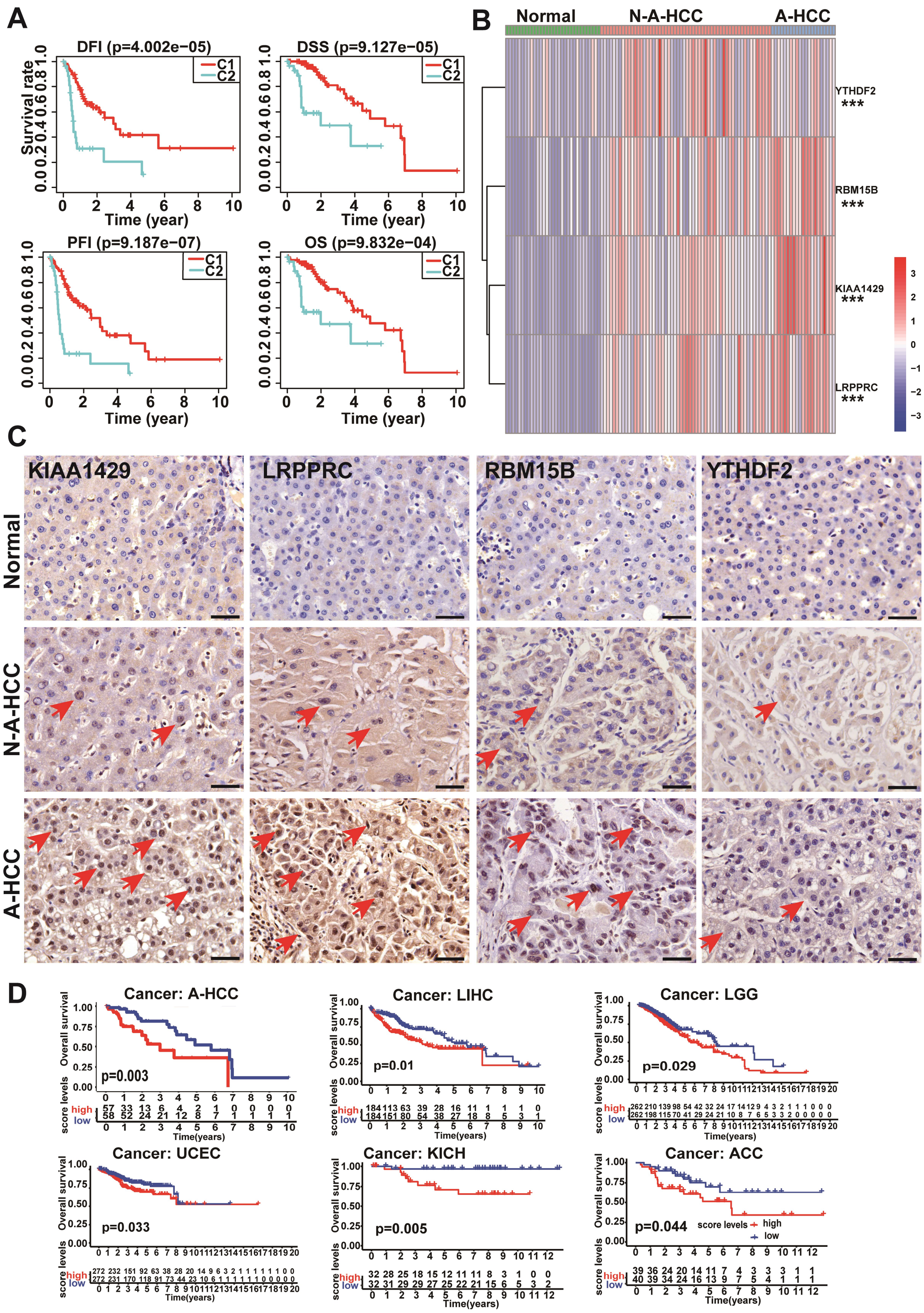

Figure S1. External applicability of the m6A model

(A) Figure 3G different survival intervals (DFI/DSS/PFI/OS) of patients in two clusters.

(B-C) The qRT-PCR expression (B) and immunohistochemical staining(C) of KIAA1429/LRPPRC/RBM15B/YTHDF2 in clinical patients of three groups was observed: Normal (n = 31), N-A-HCC (no history of alcohol consumption n = 56), and A-HCC (n = 21). (D) The m6A model was predictive of multiple tumour survival: A-HCC (P=0.003). Liver hepatocellular carcinoma (LIHC, P=0.01), Lower Grade Glioma (LGG, P=0.029), Uterine Corpus Endometrial Carcinoma (UCEC, P=0.033) KIDNEY Chromophobe (KICH, P=0.005) and Arenal cortical Carcinoma (ACC, P=0.044).

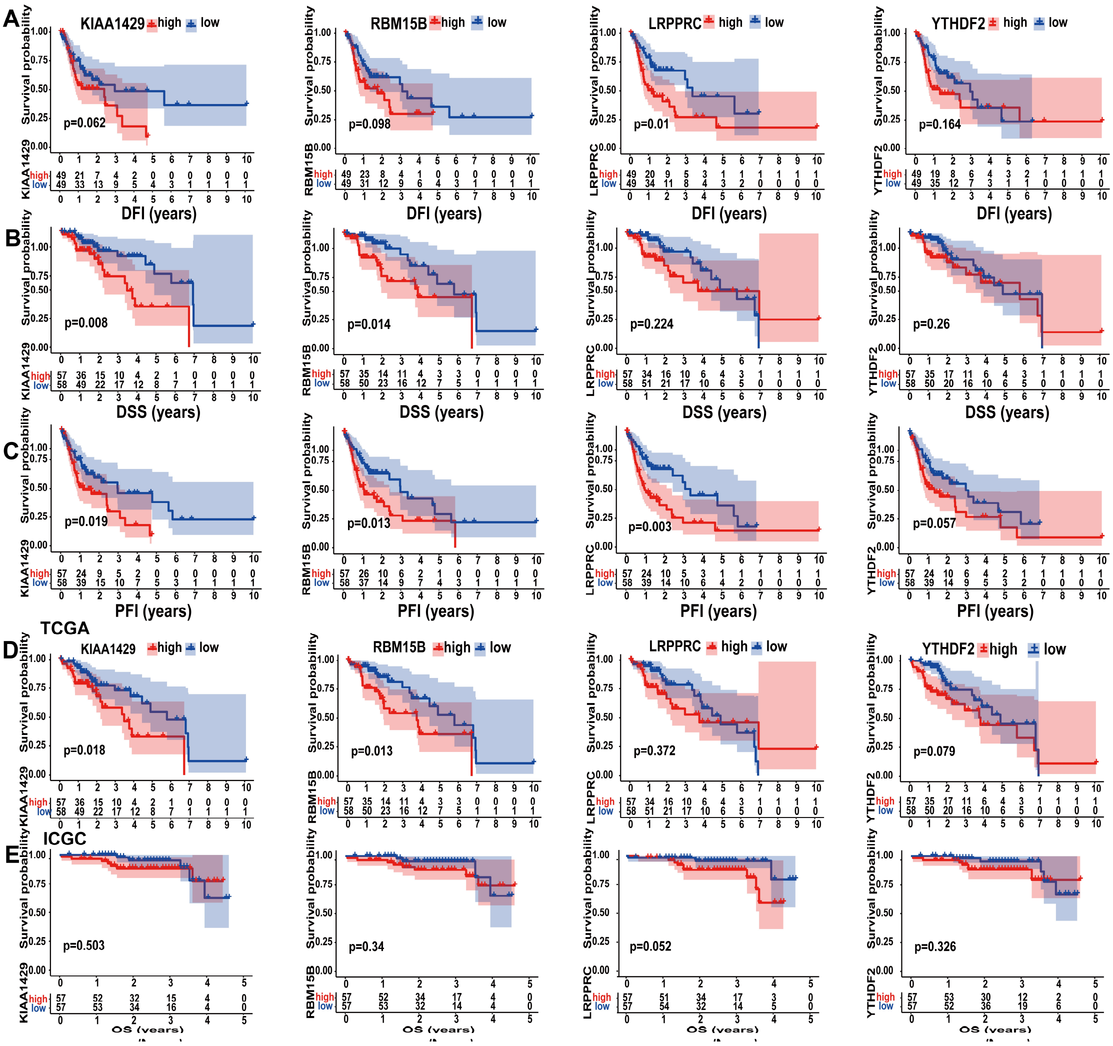

Figure S2. Kaplan–Meier analysis of different survival times in the TCGA-A-HCC cohort

(A) Different factors (*KIAA1429, LRPPRC, RBM15B,* and *YTHDF2*) of Kaplan–Meier analysis for disease-free interval (DFI);

(B) Different factors (*KIAA1429, LRPPRC, RBM15B,* and *YTHDF2*) of Kaplan–Meier analysis for disease-specific survival (DSS);

(C) Different factors (*KIAA1429, LRPPRC, RBM15B,* and *YTHDF2*) of Kaplan–Meier analysis for progression-free survival (PFI);

(D/E) Different factors (*KIAA1429, LRPPRC, RBM15B,* and *YTHDF2*) of Kaplan–Meier analysis for overall survival (OS) in TCGA (D)/ICGC (E) databases.

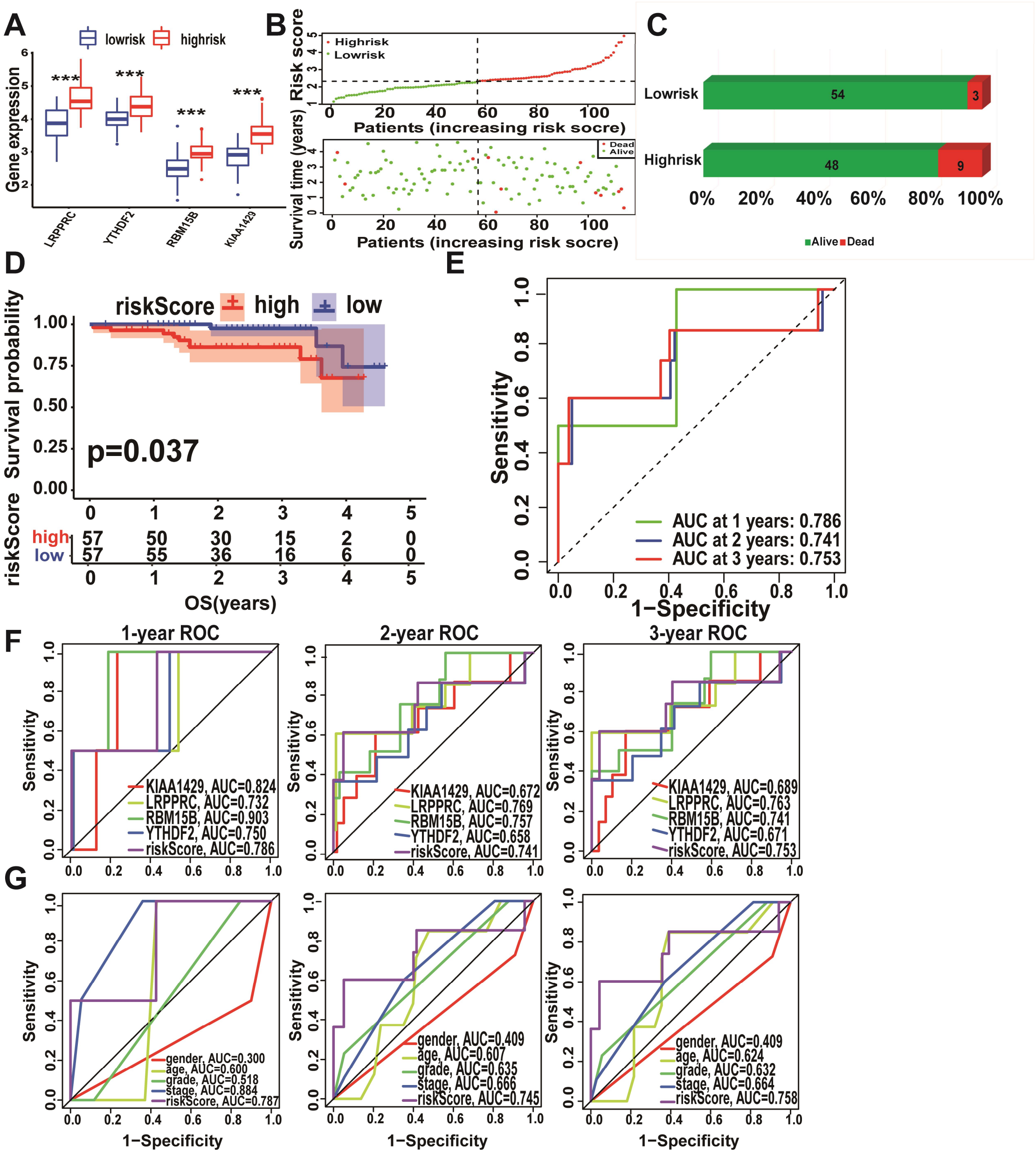

Figure S3. Performance of the m6A-risk model in predicting A-HCC patient survival in ICGC databases.

(A) Boxplots showing four m6A-related gene expression profiles in high-risk and low-risk subtypes.

(B) Patient status distribution in the high-risk and low-risk subtypes.

(C) Mortality rates of the high-risk and low-risk subtypes.

(D) Overall survival curves for A-HCC patients.

(E-G) ROC curves of TCGA cohort: ROC curves showing the predictive accuracy of model (E)/model-related genes (F)/different clinical characteristics and time (1/2/3 year) (G).

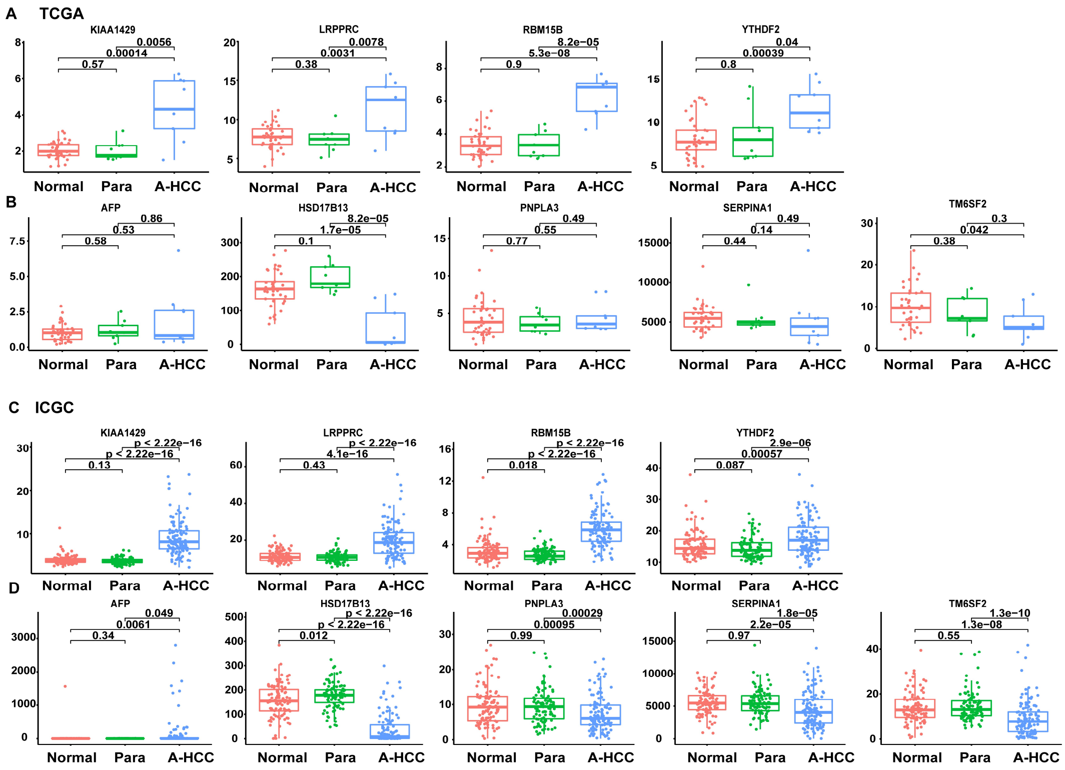

Figure S4. Difference among normal individuals and paracarcinoma (para) and A-HCC patients

(A-B) Boxplots showing difference of model-related genes (A)/validated predictors (B) in TCGA databases.

(C-D) Boxplots showing difference of model-related genes (C)/validated predictors (D) in ICGC databases.

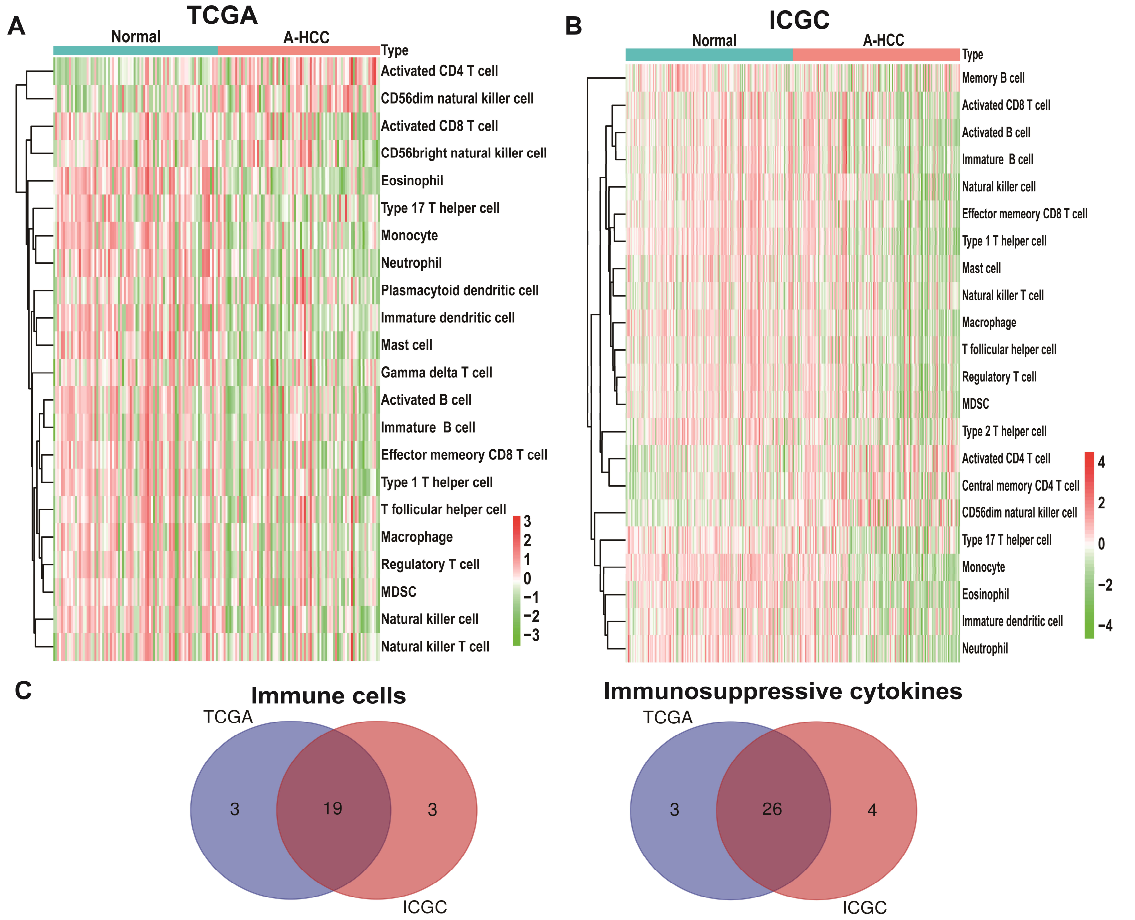

Figure S5. Immune microenvironment prediction

(A) Relative proportion of immune cell infiltration in all TCGA-A-HCC patients.

(B) Relative proportion of immune cell infiltration in all ICGC-A-HCC patients.

(C) Venn diagram of overlapping immune cells and immunosuppressive cytokines between TCGA and ICGC databases.

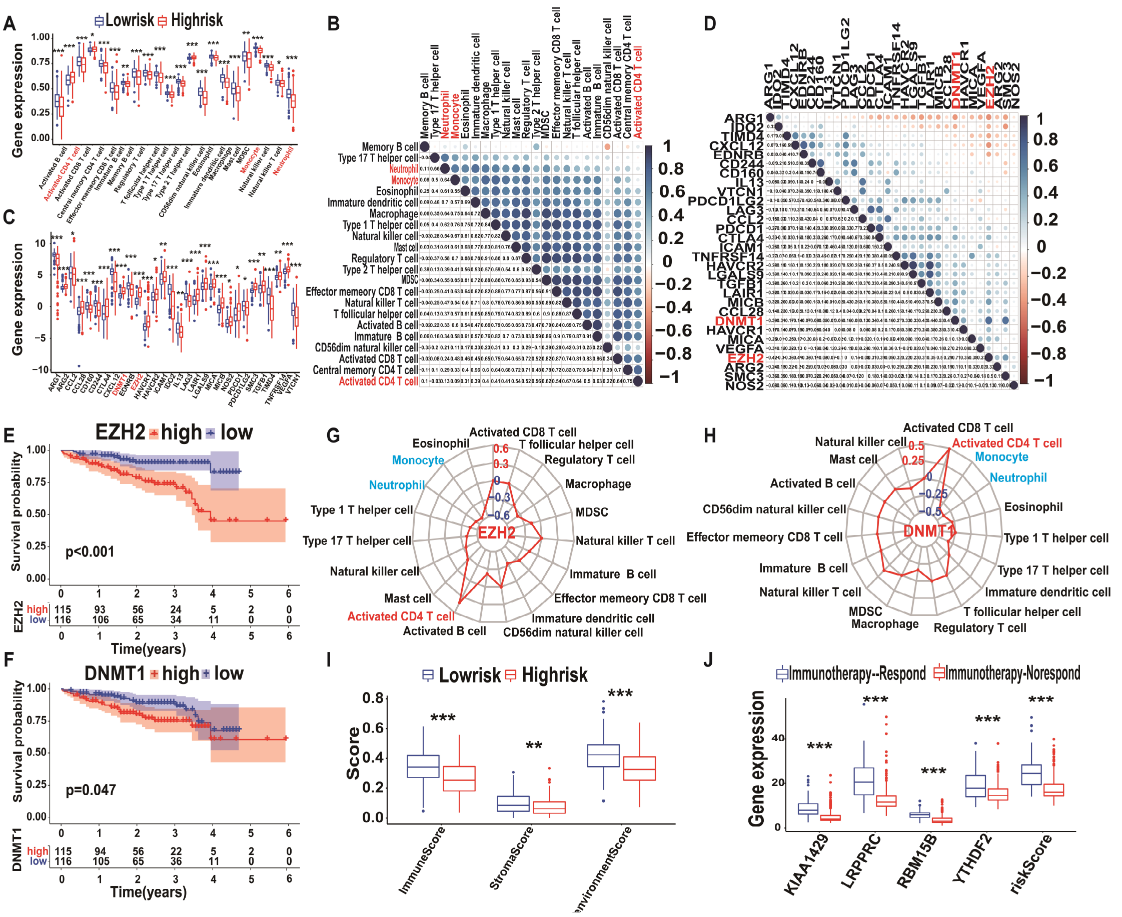

Figure S6. Immune landscape and immunotherapy prediction between low and high m6A-risk A-HCC patients in ICGC databases.

(A) Boxplot visualizing the difference of immune cell infiltration among different risk subtypes from ICGC-A-HCC. * P < 0.05, ** P < 0.01, *** P < 0.001.

(B) Correlation analysis of immune cells from ICGC-A-HCC.

(C) Boxplot visualizing the different expression of immunosuppressive cytokines among different risk subtypes from ICGC-A-LIHC. * P < 0.05, ** P < 0.01, *** P < 0.001.

(D) Correlation analysis of immunosuppressive cytokines from ICGC-A-HCC.

(E-F) Kaplan–Meier analysis of DNMT1 (E) and EZH2 (F) for OS between different risk subtypes.

(G-H) Radar map showing relationship between immune cells and DNMT1 (G)/EZH2 (H).

(I) Boxplot of the relationship between ImmuneScore StromaScore ImmuneScore/StromaScore-MicroenvironmentScore.

(J) Boxplot showing risk scores and four hub genes (KIAA1429, LRPPRC, RBM15B, and YTHDF2) between the immunotherapy non-response and immunotherapy response groups.

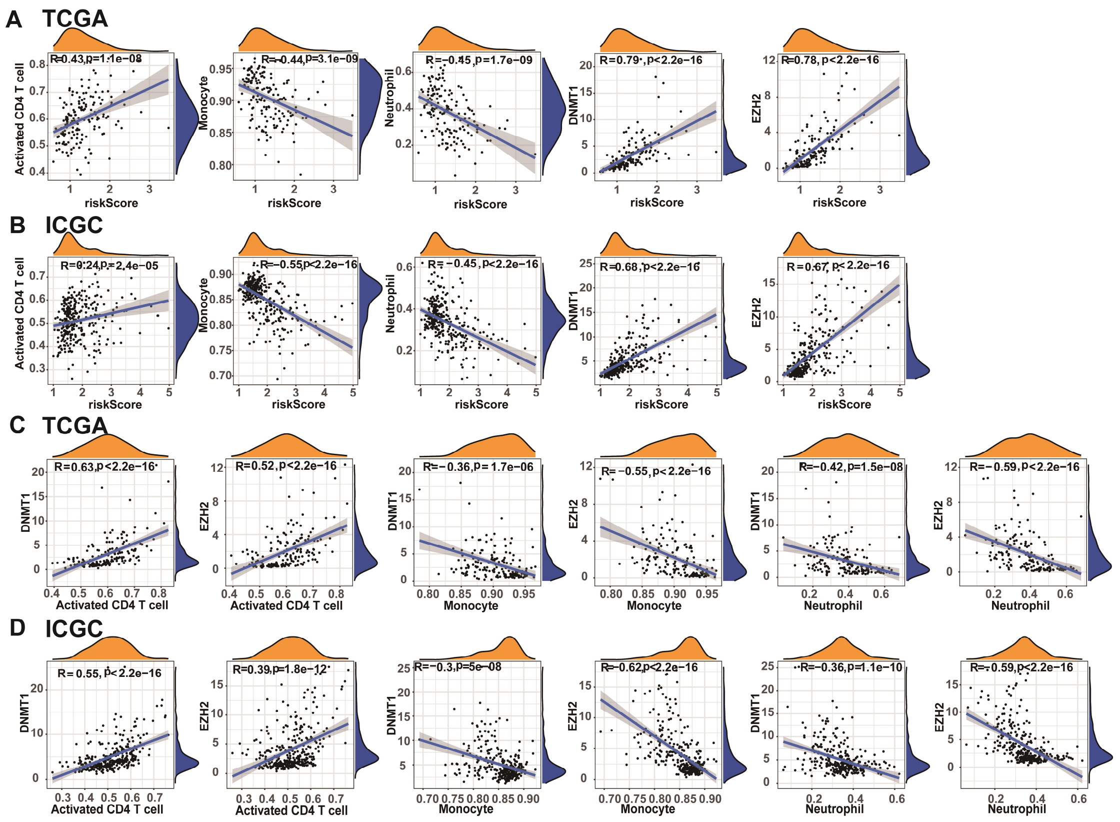

Figure S7. Correlation between risk scores, immune cells, and immunosuppressive cytokines

(A-B) Diagram showing the correlation between risk scores and activated CD4+ T cell/monocyte/neutrophil/DNMT1/EZH2 (from left to right) in TCGA (A) and ICGC (B) databases.

(C-D) Diagram showing the correlation between immune cells (Activated CD4+ T cell/monocyte/neutrophil) and immunosuppressive cytokines (DNMT1/EZH2) in TCGA (C) and ICGC (D) databases.

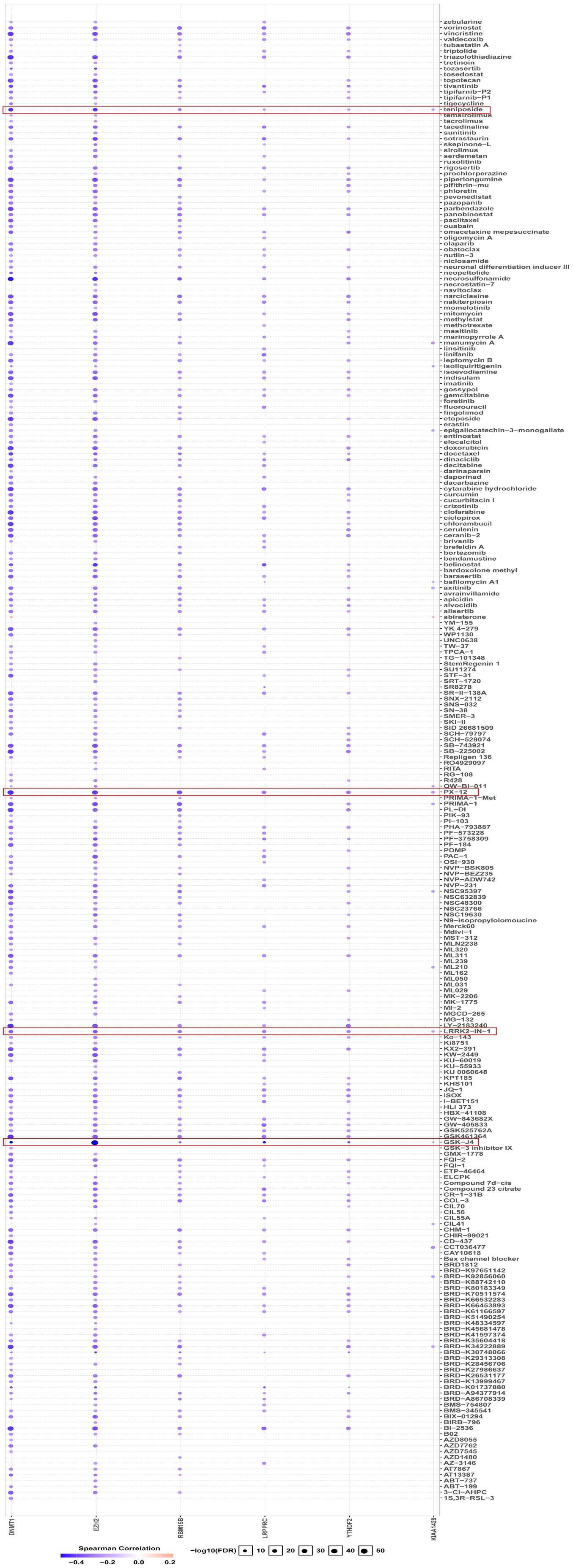

zebularine
vorinostat
vincristine
valdecoxib
tubastatin A
triptolide
triazolothiadiazine
tretinoin
tozasertib
tosedostat
topotecan
tivantinib
tipifarnib−P2
tipifarnib−P1
tigecycline
teniposide
temsirolimus
tacrolimus
tacedinaline
sunitinib
sotrastaurin
skepinone−L
sirolimus
serdemetan
ruxolitinib
rigosertib
prochlorperazine
piperlongumine
pifithrin−mu
phloretin
pevonedistat
pazopanib
parbendazole
panobinostat
paclitaxel
ouabain
omacetaxine mepesuccinate
oligomycin A
olaparib
obatoclax
nutlin−3
niclosamide
neuronal differentiation inducer III
neopeltolide
necrosulfonamide
necrostatin−7
navitoclax
narciclasine
nakiterpiosin
momelotinib
mitomycin
methylstat
methotrexate
masitinib
marinopyrrole A
manumycin A
linsitinib
linifanib
leptomycin B
isoliquiritigenin
isoevodiamine
indisulam
imatinib
gossypol
gemcitabine
foretinib
fluorouracil
fingolimod
etoposide
erastin
epigallocatechin−3−monogallate
entinostat
elocalcitol
doxorubicin
docetaxel
dinaciclib
decitabine
darinaparsin
daporinad
dacarbazine
cytarabine hydrochloride
curcumin
cucurbitacin I
crizotinib
clofarabine
ciclopirox
chlorambucil
cerulenin
ceranib−2
brivanib
brefeldin A
bortezomib
bendamustine
belinostat
bardoxolone methyl
barasertib
bafilomycin A1
axitinib
avrainvillamide
apicidin
alvocidib
alisertib
abiraterone
YM−155
YK 4−279
WP1130
UNC0638
TW−37
TPCA−1
TG−101348
StemRegenin 1
SU11274
STF−31
SRT−1720
SR8278
SR−II−138A
SNX−2112
SNS−032
SN−38
SMER−3
SKI−II
SID 26681509
SCH−79797
SCH−529074
SB−743921
SB−225002
Repligen 136
RO4929097
RITA
RG−108
R428
QW−BI−011
PX−12
PRIMA−1−Met
PRIMA−1
PL−DI
PIK−93
PI−103
PHA−793887
PF−573228
PF−3758309
PF−184
PDMP
PAC−1
OSI−930
NVP−BSK805
NVP−BEZ235
NVP−ADW742
NVP−231
NSC95397
NSC632839
NSC48300
NSC23766
NSC19630
N9−isopropylolomoucine
Merck60
Mdivi−1
MST−312
MLN2238
ML320
ML311
ML239
ML210
ML162
ML050
ML031
ML029
MK−2206
MK−1775
MI−2
MGCD−265
MG−132
LY−2183240
LRRK2−IN−1
Ko−143
Ki8751
KX2−391
KW−2449
KU−60019
KU−55933
KU 0060648
KPT185
KHS101
JQ−1
ISOX
I−BET151
HLI 373
HBX−41108
GW−843682X
GW−405833
GSK525762A
GSK461364
GSK−J4
GSK−3 inhibitor IX
GMX−1778
FQI−2
FQI−1
ETP−46464
ELCPK
Compound 7d−cis
Compound 23 citrate
CR−1−31B
COL−3
CIL70
CIL56
CIL55A
CIL41
CHM−1
CHIR−99021
CD−437
CCT036477
CAY10618
Bax channel blocker
BRD1812
BRD−K97651142
BRD−K92856060
BRD−K88742110
BRD−K80183349
BRD−K70511574
BRD−K66532283
BRD−K66453893
BRD−K61166597
BRD−K51490254
BRD−K48334597
BRD−K45681478
BRD−K41597374
BRD−K35604418
BRD−K34222889
BRD−K30748066
BRD−K29313308
BRD−K28456706
BRD−K27986637
BRD−K26531177
BRD−K13999467
BRD−K01737880
BRD−A94377914
BRD−A86708339
BMS−754807
BMS−345541
BIX−01294
BIRB−796
BI−2536
B02
AZD8055
AZD7762
AZD7545
AZD1480
AZ−3146
AT7867
AT13387
ABT−737
ABT−199
3−Cl−AHPC
1S,3R−RSL−3
DNMT1
EZH2
RBM15B
LRPPRC
YTHDF2
KIAA1429
Spearman Correlation
−0.4
−0.2
0.0
0.2
−log10(FDR)
10
20
30
40
50

Figure S8. Prediction of drug sensitivity for A-HCC patients

Table 1 Clinicopathological factors and the number of patients for two types of hepatocellular carcinoma, and normal subjects involved in this study

		**N-A-HCC**	**A-HCC**	**Normal**
**Characteristic**	**Category**	**Number of subjects**	**Number of subjects**	**Number of subjects**
**Total**		**56**	**21**	**31**
Gender	Female	21	7	12
	Male	35	14	19
Age(years)	<65	26	9	23
	≥65	30	12	8
Stage	I	23	5	
	II	15	7	
	III	18	9	
Grade	1	16	2	
	2	8	5	
	3	13	6	
	4	19	8	
Status	Alive	52	18	31
	Dead	4	3	0

Table 2 Primers included in the RT-qPCR assay.

**Primer**	**5'→3' Sequence**
Dnmt1-F	AGGCGGCTCAAAGATTTGGAA
Dnmt1-R	GCAGAAATTCGTGCAAGAGATTC
EZH2-F	TGCAGTTGCTTCAGTACCCATAAT
EZH2-R	ATCCCCGTGTACTTTCCCATCATAAT
KIAA1429-F	CTTGGCAAGTGGCTTGAACC
KIAA1429-R	ACGTAAGGCAGTGGTAAGGC
LRPPRC-F	AGCCTGCTCCTGTGAGAAAG
LRPPRC-R	TCCCAGATCTTGTGAGCAAA
RBM15B-F	GAGAACCACTCCAGTGAAGGG
RBM15B-R	GCTGACTGGAGGTACTGCTG
YTHDF2-F	TAGCCAACTGCGACACATTC
YTHDF2-R	CACGACCTTGACGTTCCTTT
GAPDH-F	CCCACTCCTCCACCTTTGAC
GAPDH-R	TCCTCTTGTGCTCTTGCTGG

Table 3 Characters of ALD-HCC patients in TCGA

Characteristic	Category	Number of patients
Gender	Female	14
	Male	103
Age(years)	<65	64
	≥65	53
Stage	I	55
	II	21
	III	31
Grade	1	18
	2	58
	3	39
	4	2
Status	Alive	79
	Dead	38
Adjacent tissue inflammation	Mild	20
	Severe	5
	Other	92
Fibrosis	0-2	27
	3-4	9
	5-6	16
	Other	65

Table 4 Characters of ALD-HCC patients in ICGA

Characteristic	Category	Number of patients
Gender	Female	13
	Male	101
Age(years)	<65	47
	≥65	67
Grade	I	16
	II	83
	III	12
Stage	1	20
	2	50
	3	36
	4	8
Status	Alive	102
	Dead	12
fibrosis	0-2	52
	3-4	62
Tumor venous Infiltration	Yes	34
	No	77

Table 5 UnivariateCox Analysis

gene	HR	z	pvalue
YTHDF2	4.528899	3.333443	0.000858
KIAA1429	2.865334	2.913846	0.00357
YTHDF1	3.147081	2.719835	0.006531
RBM15B	2.61164	2.599653	0.009332
LRPPRC	2.296597	2.488739	0.01282
RBM15	2.598275	2.410586	0.015927
YTHDF3	1.935591	2.105286	0.035266
HNRNPC	1.789915	1.745728	0.080858
WTAP	1.528766	1.308472	0.190713
IGF2BP1	1.21253	1.269264	0.204347
METTL3	1.475177	1.185321	0.235891
HNRNPA2B1	1.335914	0.952523	0.340832
YTHDC1	1.361391	0.840087	0.400859
ELAVL1	1.397328	0.821852	0.411161
ALKBH5	0.793244	-0.76873	0.442053
CBLL1	1.233887	0.675424	0.499406
FTO	0.78936	-0.5728	0.566777
ZC3H13	0.880681	-0.54288	0.587214
METTL14	1.171621	0.410549	0.681403
YTHDC2	0.936664	-0.19145	0.848171
FMR1	1.005043	0.021778	0.982625

Table 6 Clinical survival in both subtypes(C1/C2)

Type		C1				C2			
DFI	Time (y)	Paients (n)	Survival	SE	95% CI	Paients (n)	Survival	SE	95% CI
	1	50	0.774	0.0515	0.679-0.882	8	0.308	0.095	0.1685-0.564
	3	12	0.509	0.0837	0.368-0.702	3	0.206	0.1051	0.0754-0.56
	5	10	0.416	0.0905	0.272-0.637	2	0.103	0.0897	0.0186-0.569
DSS	1	79	0.987	0.0126	0.963-1	15	0.591	0.1001	0.424-0.824
	3	24	0.777	0.0663	0.6569-0.918	6	0.493	0.1227	0.302-0.803
	5	10	0.548	0.1014	0.3815-0.788	3	0.328	0.1571	0.129-0.839
PFI	1	59	0.764	0.0474	0.677-0.863	7	0.2352	0.0837	0.1172-0.472
	3	14	0.451	0.0769	0.3225-0.629	3	0.1568	0.0849	0.0543-0.453
	5	6	0.318	0.0878	0.1848-0.546	2	0.0784	0.0698	0.0137-0.449
OS	1	79	0.9519	0.0235	0.9071-0.999	15	0.568	0.0988	0.404-0.798
	3	24	0.7181	0.0666	0.5987-0.861	6	0.473	0.1193	0.288-0.776
	5	10	0.4787	0.0943	0.3254-0.704	3	0.315	0.1513	0.123-0.808

Table 7 GSEA analysis

NAME	TCGA				ICGC		
	SIZE	ES	NES	NOM p-val	SIZE	ES	NES
G2M_CHECKPOINT	195	0.773925	1.872758	<0.001	190	0.722994	2.002184
PI3K_AKT_MTOR_SIGNALING	105	0.654883	1.894131	<0.001	104	0.536363	1.941547
PROTEIN_SECRETION	96	0.70774	1.958296	<0.001	95	0.641883	2.105106
UNFOLDED_PROTEIN_RESPONSE	110	0.646417	1.91497	<0.001	106	0.584688	1.963052
MYC_TARGETS_V1	196	0.711536	1.799156	0.001996	193	0.722594	1.949364
E2F_TARGETS	198	0.752525	1.788301	0.003976	194	0.756297	2.03516
MTORC1_SIGNALING	196	0.590905	1.851324	0.004016	194	0.601345	2.022227
DNA_REPAIR	149	0.625902	1.837142	<0.001	141	0.562544	1.937359
MITOTIC_SPINDLE	198	0.762504	1.998572	<0.001	197	0.576724	1.898549
ANDROGEN_RESPONSE	98	0.511055	1.651018	0.00616	96	0.446966	1.724436
HEME_METABOLISM	193	0.533672	1.821891	<0.001	191	0.373494	1.591133
GLYCOLYSIS	197	0.507472	1.772849	0.002092	197	0.443539	1.760442
SPERMATOGENESIS	133	0.522367	1.701034	0.004132	131	0.44227	1.576259
UV_RESPONSE_UP	156	0.497127	1.693498	0.002045	152	0.352935	1.485076
WNT_BETA_CATENIN_SIGNALING	42	0.684786	1.79914	<0.001	42	0.46991	1.579675

Table 8 Relationship between DNMT1/EZH2 and immune cells in TCGA/ICGC database

Immune Cell	EZH2		DNMT1	
	ICGC	TCGA	ICGC	TCGA
Activated CD4 T cell	0.41	0.49	0.49	0.56
Monocyte	-0.56	-0.45	-0.35	-0.39
Neutrophil	-0.52	-0.44	-0.38	-0.36
Activated CD8 T cell	0	-0.17	0	0
Eosinophil	-0.44	-0.51	-0.29	-0.33
Type 1 T helper cell	-0.46	-0.42	-0.25	-0.18
Type 17 T helper cell	-0.41	-0.31	-0.33	-0.2
Natural killer cell	-0.35	-0.35	-0.11	0
Mast cell	-0.26	-0.48	0	-0.31
Activated B cell	-0.22	-0.25	0	0
CD56dim natural killer cell	0	0.22	0	0.27
Immature dendritic cell	-0.34	-0.38	-0.21	-0.17
Effector memeory CD8 T cell	-0.24	-0.35	0	0
Immature B cell	-0.17	-0.22	0	0
Natural killer T cell	0	-0.2	0.12	0
MDSC	-0.17	-0.18	0	0
Macrophage	-0.36	-0.24	-0.16	0
Regulatory T cell	-0.27	-0.22	0	0
T follicular helper cell	0	0	0	0

Table 9 m6A regulatory gene and associated pathway

Gene_Name	Locus	Trans_Type	p_value	Pathway
ABCA3	chr16:2325882-2390736	protein_coding	0.0255192	Regulation of activated PAK-2p34 by proteasome mediated degradation;Metabolism of proteins;Surfactant metabolism; Transport of glucose and other sugars, bile salts and organic acids, metal ions and amine compounds
	chr16:2325882-2390747	protein_coding	0.0100588	
	chr16:2373340-2379725	protein_coding	0.4968346	
ACTA2	chr10:90694831-90712530	protein_coding	0.6751824	Actin Nucleation by ARP-WASP Complex;PAK Pathway;VEGF Pathway; fMLP Pathway;Sertoli-Sertoli Cell Junction Dynamics
	chr10:90701542-90751096	protein_coding	0.0014677	

ATP1A3	chr10:90701542-90751045	protein_coding	0.0224629	Cardiac conduction;Aldosterone synthesis and secretion;cGMP-PKG signaling pathway;Salivary secretion;Aldosterone-regulated sodium reabsorption
	chr19:42470734-42498428	protein_coding	0.0495627	
	chr19:42470773-42497619	protein_coding	0.0040896	
	chr19:42470736-42498412	protein_coding	0.4422272	
	chr19:42470736-42498231	protein_coding	0.5516858	
C7	chr5:40909354-40983041	protein_coding	0.0044536	Immune response Lectin induced complement pathway;Complement Pathway; Complement and coagulation cascades; Creation of C4 and C2 activators;Innate Immune System
	chr5:40936440-40937589	processed_transcript	0.4955184	
CEND1	chr11:787104-790123	protein_coding	0.0013529	DNA Damage;Neuroscience
CERS4	chr19:8274237-8327305	protein_coding	0.0333834	Sphingolipid metabolism;Metabolism;Sphingolipid signaling pathway
	chr19:8274197-8327305	protein_coding	0.0395157	
	chr19:8289911-8321559	protein_coding	0.5554278	
	chr19:8274255-8327305	protein_coding	0.7150772	
	chr19:8274240-8327305	protein_coding	0.2069862	
	chr19:8274518-8316055	protein_coding	0.5644867	
	chr19:8274210-8320608	protein_coding	0.9928841	
	chr19:8274267-8327305	processed_transcript	0.4408055	
COL13A1	chr10:71561688-71718904	protein_coding	0.0011521	Collagen chain trimerization;Integrin Pathway;ERK Signaling;

	chr10:71561688-71718904	protein_coding	0.0336758	Degradation of the extracellular matrix;Phospholipase-C Pathway
	chr10:71561874-71718489	protein_coding	0.6720126	
	chr10:71690209-71718683	protein_coding	0.4893448	
	chr10:71637052-71639183	processed_transcript	0.1142819	
	chr10:71703881-71724031	processed_transcript	0.4189301	
	chr10:71562180-71718457	protein_coding	0.5290397	
	chr10:71561874-71718489	protein_coding	0.7242527	
CYB5R2	chr11:7686331-7694732	protein_coding	0.0227442	O2/CO2 exchange in erythrocytes;Amino sugar and nucleotide sugar metabolism;PAK Pathway; Metabolism;Cytochrome P450 - arranged by substrate type
	chr11:7690436-7698453	protein_coding	0.8202211	
	chr11:7686331-7694703	protein_coding	0.2063338	
	chr11:7689743-7695449	protein_coding	0.7598926	
	chr11:7686335-7690548	processed_transcript	0.5220367	
	chr11:7686335-7695439	protein_coding	0.3905512	
	chr11:7686365-7688991	processed_transcript	0.0006005	
DYSF	chr2:71693832-71913778	protein_coding	0.164561	Cardiac conduction;Smooth Muscle Contraction
	chr2:71693832-71913778	protein_coding	0.5256073	
	chr2:71680852-71913778	protein_coding	0.4226497	
	chr2:71693832-71913778	protein_coding	0.177521	

	chr2:71693832-71913778	protein_coding	0.164103	
	chr2:71680852-71913778	protein_coding	0.4226497	
	chr2:71693832-71913778	protein_coding	0.0417304	
	chr2:71693832-71913778	protein_coding	0.261523	
	chr2:71680852-71913778	protein_coding	0.4226497	
	chr2:71680852-71913778	protein_coding	0.4226497	
	chr2:71804029-71913893	processed_transcript	0.0023687	
ELOVL6	chr4:110970543-111119832	protein_coding	0.0246844	Fatty Acyl-CoA Biosynthesis;Metabolism;alpha-linolenic (omega3) and linoleic (omega6) acid metabolism; Regulation of cholesterol biosynthesis by SREBP (SREBF);Regulation of lipid metabolism by Peroxisome proliferator-activated receptor alpha (PPARalpha)
	chr4:110967002-111119809	protein_coding	0.0565042	
	chr4:111119047-111119522	processed_transcript	0.7084527	
ETV1	chr7:13932655-14029301	protein_coding	0.0022337	Transcriptional misregulation in cancer; MAPK Erk Pathway;P38 MAPK Signaling Pathway (sino); Akt Signaling;Development_TGF-beta receptor signaling
	chr7:13934006-14029572	protein_coding	0.1248061	
	chr7:13930853-14029571	protein_coding	0.7347311	
	chr7:14027784-14029291	protein_coding	0.2127708	
	chr7:14014489-14029264	processed_transcript	0.0353355	
FAM213B	chr1:2518237-2522902	protein_coding	0.0150895	Metabolism; Arachidonic acid metabolism;Linoleic acid metabolism
	chr1:2518214-2522907	protein_coding	0.5501158	
	chr1:2518272-2520416	protein_coding	0.2233106	
	chr1:2518481-2520952	processed_transcript	0.392872	

FMN2	chr1:240255180-240638489	protein_coding	0.3005904	Cellular response to DNA damage stimulus;Wnt-Associated β-Catenin pathway
	chr1:240408560-240492692	protein_coding	0.9323489	
	chr1:240492444-240638483	protein_coding	0.0049431	
ITGB4	chr17:73717516-73753899	protein_coding	0.6485075	Apoptotic Pathways in Synovial Fibroblasts;ERK Signaling;Integrin Pathway;Focal Adhesion;Actin Nucleation by ARP-WASP Complex
	chr17:73720776-73753899	protein_coding	0.0224685	
	chr17:73717408-73753685	protein_coding	0.7789932	
	chr17:73720784-73738738	processed_transcript	0.002448	
	chr17:73750699-73753083	protein_coding	0.1905888	
KIF3C	chr2:26149465-26205366	protein_coding	0.0081429	Golgi-to-ER retrograde transport;Organelle biogenesis and maintenance;Vesicle-mediated transport;Class I MHC mediated antigen processing and presentation;Factors involved in megakaryocyte development and platelet production
	chr2:26151622-26205618	protein_coding	0.6248069	
	chr2:26152288-26174788	processed_transcript	0.8465347	
LUM	chr12:91496406-91505608	protein_coding	0.0046983	Defective B4GALT1 causes B4GALT1-CDG (CDG-2d);Keratan sulfate/keratin metabolism;3 Glycosaminoglycan metabolism; Proteoglycans in cancer;HIV Life Cycle
MAGED1	chrX:51636698-51645453	protein_coding	0.3755072	p75 NTR receptor-mediated signalling;Development HGF signaling pathway;Apoptotic
	chrX:51636735-51645450	protein_coding	0.0253306	

	chrX:51636629-51645450	protein_coding	0.0608061	execution phase;CASP8 activity is inhibited;Signaling by GPCR
	chrX:51546103-51645450	protein_coding	0.0050974	
	chrX:51636742-51638848	processed_transcript	0.4150908	
	chrX:51637665-51638798	processed_transcript	0.7410387	
	chrX:51638752-51639570	processed_transcript	0.0420194	
	chrX:51636754-51645448	processed_transcript	0.9855295	
	chrX:51636745-51645450	processed_transcript	0.0202818	
MFGE8	chr15:89441945-89456642	protein_coding	0.2702348	Activated PKN1 stimulates transcription of AR (androgen receptor) regulated genes KLK2 and KLK3; Metabolism of proteins; Extracellular vesicle-mediated signaling in recipient cells;Integrins in angiogenesis
	chr15:89441944-89456612	protein_coding	0.6270374	
	chr15:89442546-89456610	protein_coding	0.22527	
	chr15:89444782-89456593	protein_coding	0.2866173	
	chr15:89448630-89456610	processed_transcript	0.2154945	
	chr15:89447021-89448159	processed_transcript	0.0406788	
	chr15:89441946-89456612	protein_coding	0.0078043	
	chr15:89445414-89447877	processed_transcript	0.2611109	
OLR1	chr12:10310900-10324737	protein_coding	0.0120261	Innate Immune System;Phagosome;PPAR signaling pathway;Cell surface interactions at the vascular wall;Response to elevated platelet cytosolic Ca2+
	chr12:10313477-10322986	protein_coding	0.2604333	

RASD2	chr22:35936915-35950048	protein_coding	0.031554	cAMP/PKA-dependent signaling pathway;Mtor1 pathway
SPEG	chr2:220299568-220358354	protein_coding	0.0065073	Muscle organ development;Negative regulation of cell proliferation
	chr2:220306767-220331582	protein_coding	0.0297217	
	chr2:220306745-220313237	protein_coding	0.4954399	
	chr2:220310349-220358315	processed_transcript	0.0011973	
	chr2:220310302-220313177	processed_transcript	0.4843369	
	chr2:220301997-220331583	processed_transcript	0.0155958	
ST6GAL1	chr3:186648274-186796341	protein_coding	0.047782	Synthesis of substrates in N-glycan biosythesis;Transport to the Golgi and subsequent modification;Metabolism of proteins;N-glycan antennae elongation in the medial/trans-Golgi;O-linked glycosylation
	chr3:186648315-186796341	protein_coding	0.1923071	
